# Understanding, controlling and optimising the cooling of waste thermal treatment beds including STARx Hottpads

**DOI:** 10.1177/0734242X221076308

**Published:** 2022-03-21

**Authors:** Ryan B Morales, Christopher T DeGroot, Grant C Scholes, Jason I Gerhard

**Affiliations:** 1 Department of Civil and Environmental Engineering, The University of Western Ontario, London, ON, Canada; 2 Department of Mechanical and Materials Engineering, The University of Western Ontario, London, ON, Canada; 3 Savron, Guelph, ON, Canada

**Keywords:** Applied smouldering systems, cooling time reduction, forced convection, hot packed bed, process optimisation, STARx, thermally induced air channelling, waste thermal treatment

## Abstract

STARx (Self-sustaining Treatment for Active Remediation ex situ) is a thermal treatment strategy for contaminated soils and organic wastes. Key to this technology is that organics are embedded in porous matrix beds (e.g. sand). STARx induces a self-sustaining smouldering combustion front that traverses the bed, burning away the embedded contaminants/wastes. The time and cost effectiveness of this technology is largely dictated by the time required for cooling of the hot, clean, porous matrix bed that remains after treatment. This study is the first to explore the cooling of these beds. A suite of novel simulations investigated the influence of key parameters on bed-cooling time. The results reveal that cooling time decreased nearly linearly with decreases of volume-averaged bed temperature and bed bulk density. Increased injection air fluxes led to the non-linear decrease of cooling time. Also, cooling time was negatively impacted by bed temperature inhomogeneity, which influenced preferential air flow through cooler regions of the bed, bypassing hotter regions. From these results, using lower bulk density bed materials, increased air fluxes and enhancing wall insulation to improve bed temperature homogeneity were identified as system optimisations to reduce cooling times. While the aim of this research is to improve the STARx cooling process, the results are also highly applicable to many similar engineering systems that involve hot porous bed cooling.

## Introduction

Smouldering combustion is an exothermic reaction in which heat and oxygen attack the surface of a condensed phase fuel (Ohlemiller, 1985). A common example of smouldering is the glowing red surface of charcoal briquettes in a barbeque. Smouldering is ignited rapidly by a localised heat flux (e.g. a fire in a coal seam, burning branch falling on peat forest floor). Through the convection and diffusion of air, oxidiser (oxygen) is delivered to the reaction site, known as the ‘smouldering front’ (Rein, 2009; Torero et al., 2020; Zanoni et al., 2019a). Heat released from the oxidation reaction in the front is transferred (via convection, conduction and radiation) to adjacent fuel (Ohlemiller, 1985). As a result, the smouldering front propagates slowly through the fuel, consuming it in the process. Smouldering fuels can be solid or liquid (Pironi et al., 2009, 2011; Switzer et al., 2009; Torero et al., 2020). However, regardless of the fuel type, a key requirement for smouldering is the presence of a porous matrix. This provides a network of paths for oxygen to reach the fuel, a high surface area for mass and heat transfer and insulation of the reaction (Rein, 2009; Torero et al., 2020; Yermán et al., 2015). For instance, when smouldering a solid fuel such as charcoal, the charcoal itself provides the porous matrix. Similarly, although liquid fuels (e.g. coal tar) are non-porous, they can be made smoulderable by mixing with a porous matrix such as quartz sand.

A key feature of smouldering is that when conditions are appropriate (e.g. sufficient fuel concentration, fuel type and air flux), the process is self-sustaining (Torero et al., 2020; Wyn et al., 2020; Zanoni et al., 2019a). This means that more energy is generated (i.e. heat evolved from the oxidation reactions) than is lost (e.g. to endothermic reactions like pyrolysis) and the front progresses without any external energy input. As such, smouldering is a highly energy-efficient process (Rashwan et al., 2021a, 2021b; Torero et al., 2020; Yermán et al., 2015). Smouldering is also self-terminating as the reaction extinguishes upon fuel depletion (Crielaard et al., 2019; Switzer et al., 2014; Wang et al., 2021).

Smouldering combustion has been researched and/or applied in a variety of contexts including fire safety (Bar-Ilan et al., 2002; Leppänen and Malaska, 2019; Rein et al., 2008; Santoso et al., 2019; Steen-Hansen et al., 2018; Torero and Fernandez-Pello, 1996; Yang and Chen, 2018), oil recovery/extraction (Akkutlu and Yortsos, 2003; Castanier and Brigham, 2003; Garon et al., 1980; Martins et al., 2010; Sennoune et al., 2012; Xia et al., 2003) and sanitation (Fabris et al., 2017; Saberi et al., 2020; Yermán et al., 2015). In addition, applied smouldering systems have also emerged as a viable thermal strategy for in situ and ex situ treatment of hydrocarbon-contaminated soils (Ding et al., 2019; Gerhard et al., 2020; Grant et al., 2016; Scholes et al., 2015; Vidonish et al., 2016) as well as for disposal of waste organic liquids and sludges (e.g. waste oil sludges, petroleum hydrocarbon products; Feng et al., 2021; Rashwan et al., 2021a; Serrano et al., 2020; Wyn et al., 2020; Yermán, 2016; Zhao et al., 2021). The ex situ variant of these systems is commercially referred to as STARx (Self-sustaining Treatment for Active Remediation ex situ; Rashwan et al., 2016; Sabadell et al., 2019; Solinger et al., 2020; Switzer et al., 2014). For treatment, liquid or sludge wastes are mixed into contaminated soils or with a porous matrix (e.g. sand) to make them smoulderable. When remediating contaminated soils, the soil provides the porous matrix.

**Figure 1.**
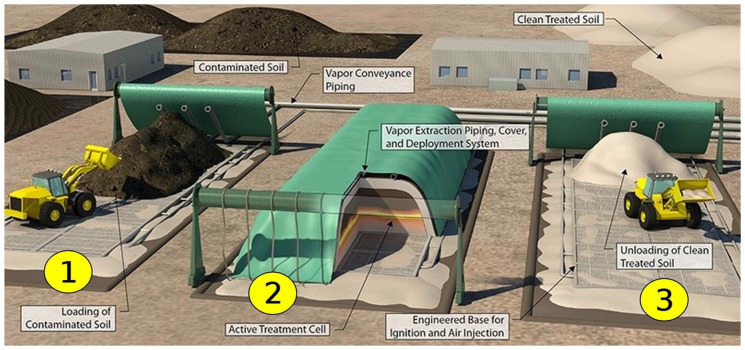
A conceptual diagram of each stage of the STARx process: (1) contaminated soil/waste loading, (2) smouldering treatment and (3) clean treated material unloading. Adapted from Murray (2019).

In the STARx process, stockpiles of contaminated soil or different porous matrix–waste mixtures are loaded on top of Hottpad^TM^ modules to form a treatment bed (Figure 1). These modules house the heating and air injection equipment used to ignite the smouldering front at the base of the bed and propagate it upwards (Solinger et al., 2020). The arrival of the front at the top of the bed indicates the end of the smouldering phase. At this point, the embedded contaminants/wastes have been burned away leaving behind only clean (i.e. carbon-free), treated bed material. STARx bed temperatures can range from 500°C to 1250°C depending on a variety of factors such as the energy content of the contaminant being oxidised. Therefore, the newly treated material must be cooled sufficiently before it can be off-loaded and replaced with more contaminated material. This portion of the treatment cycle is referred to as the ‘cooling phase’. The end of the cooling phase is designated as when the porous matrix cools to a ‘safe-to-manage’ temperature, at which point unloading commences. Figure 1 illustrates the STARx treatment system and the process progression.

Through experimentation, pilot-scale STARx technology demonstrated that a 97%–99.95% ex situ remediation efficiency was maintained for soil volumes up to 3 m^3^ (Switzer et al., 2014). Murray (2019) demonstrated the use of Hottpad^TM^ modules for ex situ remediation at scales anticipated for commercial success (> 100 m^3^). This provided the low-cost configuration of STARx (i.e. soil piles loaded on top of Hottpad^TM^ modules), which is currently used in practice. STARx Hottpads have since been commercially deployed in numerous locations worldwide including Canada, Taiwan and the USA (https://www.savronsolutions.com/).

As a thermal remediation strategy, STARx technology offers a multitude of benefits. For example, due to the self-sustaining nature of smouldering, STARx does not require consistent input of external fuel or energy (Duchesne et al., 2020; Murray, 2019). As a result, STARx is a highly energy-efficient treatment process and ranks exceptionally well on quantitative sustainability assessments (Gerhard et al., 2020). Other benefits are that STARx can be deployed as an on-site treatment process, in liquid smouldering applications the porous matrices can be reused between treatment cycles, it is easily scalable and its high degree of waste/contaminant destruction reduces environmental liabilities (Solinger et al., 2020).

Even given these advantages, process optimisations are needed to further increase effectiveness as well as reduce energy requirements, treatment times and costs. One such area for optimisation is the cycle treatment time, which can depend largely on the time required for hot post-treatment beds to cool sufficiently for unloading. Currently, cooling is carried out by continuing the air injection used previously to sustain the smouldering treatment. At present, the cooling phase duration can be similar to that of the smouldering treatment phase; for example, 6 days for treatment and a total treatment cycle time of nearly 12 days when accounting for cooling (Savron Solutions, personal communication).

Understanding and predicting how hot bed-cooling times can be reduced is needed, both for STARx and other thermal treatment technologies such as pyrolysis, incineration and gasification. However, air flow cooling of hot porous beds has not been strongly investigated. STARx research has predominately focused on the smouldering phase with no publications considering cooling phase processes or optimisations. Experiments (Laguerre et al., 2006; Pastore et al., 2018), analytical solutions (Kuznetsov, 1994, 1995, 1997; Riaz, 1978) and numerical/computational models (Kaviany, 1985; Kuwahara et al., 2000; Nijemeisland and Dixon, 2004) have focused on bed heating to investigate forced convection in porous media. Research on packed bed thermal energy storage (PBTES) systems (Cascetta et al., 2016; Sanderson and Cunningham, 1995), specifically for solar thermal energy (Gautam and Saini, 2020; Klein et al., 2014; Kuravi et al., 2013), has provided some insight into their thermal discharging. For these systems, studies have estimated bed-cooling time based on the velocity of a one-dimensional thermocline traversing the bed (McTigue et al., 2018; Torab and Beasley, 1987; White, 2011). However, this approach is not suitable for applications in which flow and heat transfer characteristics are not simple, including STARx and other solid particle processes such as coke dry quenching (CDQ) and iron ore sintering in the metallurgy sector (Jiang et al., 2019; Zhou et al., 2015).

In these cases, cooling can be subject to numerous sensitivities either unknown or with limited investigation. In particular, the influence of bed temperature inhomogeneities on cooling air flow distribution, of which there are only a few studies. Through numerical modelling of heated gas flow into a bed, several older studies identified the reduction of air flow through higher temperatures bed regions (Stanek and Szekely, 1973; Stanek and Vychodil, 1987; Vortmeyer et al., 1992; Wonchala and Wynnyckyj, 1987). More recently, Davenne et al. (2018) highlighted how the presence of a small, near-inlet, cool zone in a heated bed can substantially impact thermocline uniformity during cooling. In these few studies, the results were attributed to the temperature-dependent properties of the injected gas, but the process fundamentals or impact on bed cooling were not explored. This important system dynamic is investigated in substantial detail in this study.

Through novel simulations, this work studies the influence of key system parameters on the cooling time of hot inert porous matrix beds. This research examines the sensitivity of the cooling time to key operational variables – some within and some outside operator control – including the initial volume-averaged temperature of the bed, the temperature distribution within the bed at the start of cooling, the bulk density of the bed material and the applied injection air flux. The poorly understood thermal implications of hot porous matrix material on bed pneumatic conductivity and resulting air flow patterns are explored and explained. The impact of air flow divergence around hot zones on bed cooling is quantified. Furthermore, novel optimisation methods are provided for reducing cooling times, thereby increasing energy efficiency and reducing cost. Overall, this work provides new scientific insights as well as practical measures for a wide range of scientists and commercially active engineers.

## Methods

In this work, ANSYS^®^ Student Fluent 2020 R1/R2 was employed to simulate the cooling of heated porous matrix beds via air flow. Smouldering was not simulated, and contaminant treatment was assumed to have just completed at the start of each simulation.

### Model formulation

#### Governing equations

Air flow within the porous space was modelled as an incompressible fluid (Rashwan et al., 2021c) under fully developed, laminar conditions. The air density was determined by local temperatures at each timestep by the ideal gas law (Zanoni et al., 2017). The packed bed was assumed to be physically homogeneous and isotropic, and composed of spherical particles with an assigned intrinsic permeability $\left(k_{i}\right)$ and porosity (ϕ) . The model solved the multi-dimensional mass and momentum conservation equations in the porous bed:

(1) $$\begin{array}{c} \frac{\partial \left(\phi \rho_{g}\right)}{\partial t}+\nabla \;\cdot \;\;\left(\phi \rho_{g}{\vec{v}}_{g}\right)=0 \end{array}$$

(2) $$\begin{array}{c} {\vec{v}}_{g}=-\frac{k_{i}}{\mu_{g}}\left(\nabla p+\rho_{g}\vec{g}\right) \end{array}$$

The momentum equation, for steady flow at low Reynolds number ( Re ) with negligible macroscopic viscous effects (i.e. Brinkman term), reduces to Darcy’s law (equation (2)).

Energy conservation was governed by Fluent’s Local Thermal Non-Equilibrium Model (LTNE), which follows a dual cell approach in which spatially coincident solid and porous gas zones are defined and heat transfer is the only interaction between these zones (ANSYS Inc, 2020a). The effects of viscous dissipation on the low Re , single phase flow is assumed to be negligible. The energy equations of the gas (air) and solid (sand) phases were:

(3) $$\begin{array}{c} \begin{array}{l} \frac{\partial }{\partial t}\left(\phi \rho_{g}E_{g}\right)+\nabla \;\cdot \;\;\left(v_{g}\left(\rho_{g}E_{g}\right)\right)\\ =\nabla \;\cdot \;\;\left(\phi k_{g}\nabla T_{g}\right)+h_{sg}A_{sg}\left(T_{s}-T_{g}\right) \end{array} \end{array}$$

(4) $$\begin{array}{c} \begin{array}{l} \frac{\partial }{\partial t}\left(\left(1-\phi \right)\rho_{s}E_{s}\right)=\nabla \;\cdot \;\;\left(\left(1-\phi \right)\left[k_{rad}+k_{s}\right]\nabla T_{s}\right)\\ \;\;\;\;+h_{sg}A_{sg}\left(T_{g}-T_{s}\right) \end{array} \end{array}$$

where the interfacial area density (i.e. specific surface area) of the grains was calculated as $A_{sg}=6(1-\phi )/d_{p}$ . Radiation heat transfer was accounted for through implementation of a radiative conductivity, which followed the Rosseland approximation $\left(k_{rad}=16\sigma d_{p}T_{s}^{3}/3\right)$ (Rashwan et al., 2021c; Zanoni et al., 2017). Heat transfer between the solid and gas phase was regulated by the solid–gas heat transfer coefficient, $h_{sg}$ , estimated by Zanoni et al. (2017):

(5) $$\begin{array}{c} Nu=\frac{h_{sg}d_{p}}{k_{g}}=0.001\left(Re^{0.97}Pr^{1/3}\right) \end{array}$$

This equation is valid for Pr = 0.72, 0.125 mm ≤ $d_{p}$ ≤ 2.000 mm, and 0 ≤ Re ≤ 31, which is consistent with the cases to be presented. Moreover, this $h_{sg}$ correlation is best suited for the low Reynolds number flows applied during STARx operation (Rashwan et al., 2021c).

#### Computational domain and boundary conditions

For this study, two-dimensional (2D) grids were created to represent the vertical cross-section of a STARx treatment bed (Figure 3). Based on a field-scale system, the simulated system was 9.3 m wide and 2.5 m high. The width was implemented by creating a 4.65 m wide domain with a centreline symmetry boundary to reduce the computational cost of the simulations. The meshing procedure provided a domain of 3393 equally sized quadrilateral elements, chosen to balance accuracy with computational efficiency. Supplement A provides visualisation of the structured mesh.

For the dual cell approach, boundary conditions specific to each phase were required. For the porous gas phase, an inlet velocity boundary at the base of the bed allowed for air injected at ambient temperature $\left(T_{o}=298.15\;K\;\;\mathrm{or}\;\sim 25{}^{\circ}C\right)$ . Air exited the bed through a ‘pressure outlet’ boundary at the top of the domain, which imposes a constant static pressure and zero velocity gradient. This boundary represented the gas flow entering a hood space upon exiting the bed, reflecting commercial practice. From the hood space, air is extracted and channelled to emissions treatment systems, although this is not included in the computational domain. Although enclosed, the hood space is not sealed off, so atmospheric pressure $\left(P_{o}\right)$ was applied at the outlet. The right-side wall boundary was set as zero shear due to low Re flow (i.e. negligible velocity boundary layer effects), and zero heat flux, assuming negligible conductive heat transfer from the air to the wall. For the solid phase, the inlet and outlet were set as zero heat flux boundaries as heat transfer at these boundaries is predominantly through air convection. The right-side wall boundary was fixed at ambient temperature representing external heat loss due to lack of insulation. The left-side symmetry boundary was common to both phases. See Supplement A for a tabulated summary of all applied boundary conditions.

### Model validation and verification

**Figure 2.**
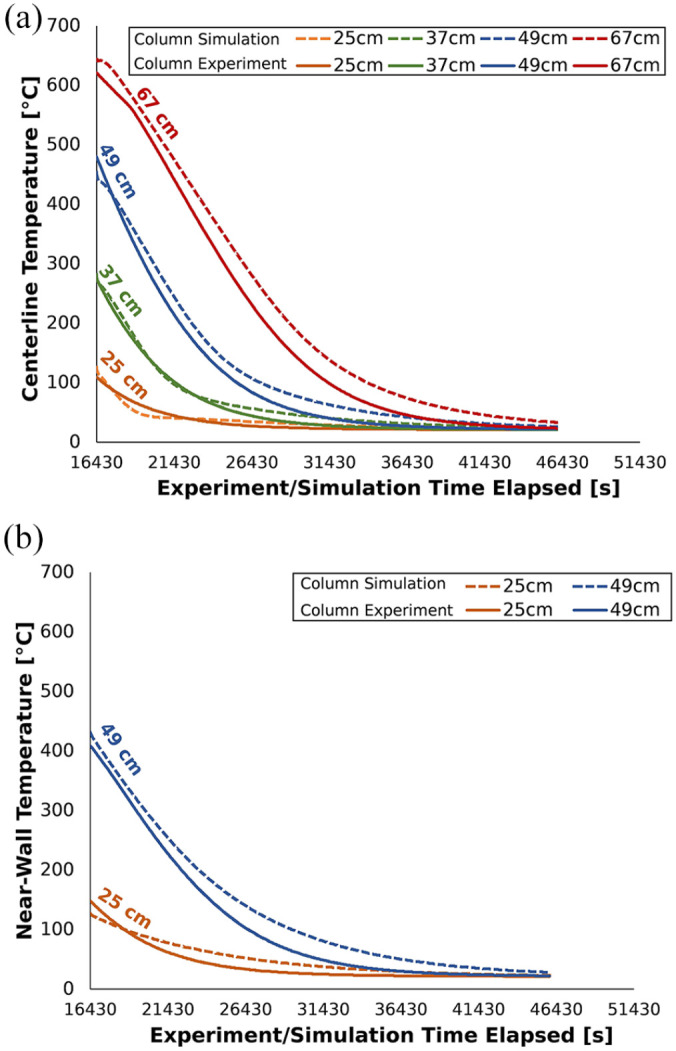
Comparison of experimental and modelled temperatures during the cooling phase. Temperatures are compared at different heights and at (a) centerline and (b) near-wall positions.

The model was validated against laboratory reactor data (Zanoni et al., 2021) during the experiment’s cooling phase (i.e. after smouldering was extinct). Temperature readings were compared at centerline and near-wall positions (i.e. 2D validation). An experiment description and the model parameters employed are provided (Supplement B). Temperatures produced from the experiment and model were compared at centerline (Figure 2(a)) and radial (i.e. near to the column; Figure 2(b)) positions.

The Normalised Root-Mean-Square Deviation between the numerical and experimental datasets was low (7%). This validation procedure provides confidence that the model properly simulates 2D packed bed cooling. In addition, it was confirmed that spatial and temporal discretisation were acceptable, and results were grid-independent (Supplement C).

### Overview of simulations

**Figure 3.**
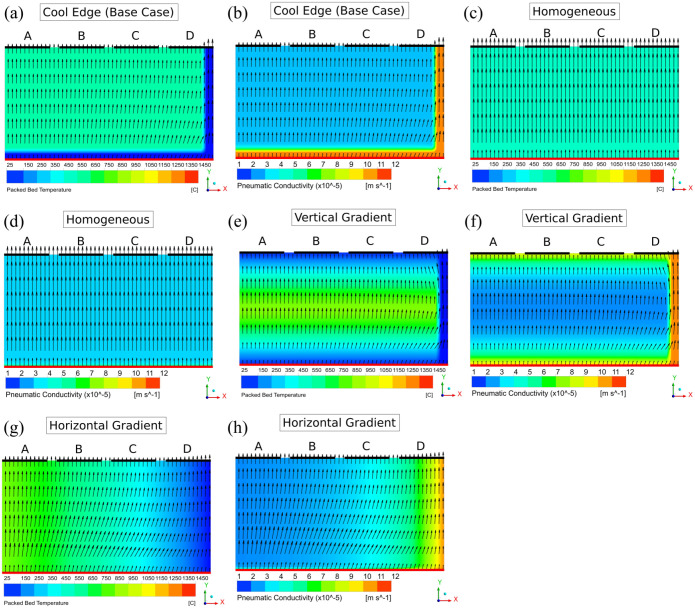
Implemented initial temperature (left-side) and resulting pneumatic conductivity (right-side) distributions: (a) and (b) Cool Edge (Base Case)], (c) and (d) Homogeneous, (e) and (f) Vertical Gradient and (g) and (h) Horizontal Gradient. The position of sampling locations A, B, C and D are indicated (black lines, top of each domain) as well as the inlet/ignition location (red line, bottom of each domain).

First, a Base Case simulation was created which employed a packed bed of medium-grained sand (mean particle diameter, $d_{p}$ , of 0.5125 mm) with its properties experimentally determined (Zanoni et al., 2017); this is hereafter referred to as ‘Base Case sand’. The Base Case injection air flux was set at 1 cm s^−1^ as commonly used in STARx systems. The bed was initialised with the ‘Cool Edge’ temperature distribution (Figure 3(a)) in which the domain was homogeneously initialised at 562°C except for 0.2 m wide near-wall and near-inlet ‘strips’, which were initialised at 40°C. These lower temperature strips reflect the influence of heat losses occurring during treatment and temperatures are representative of those seen in practice at the start of the cooling phase. Overall, the initial volume-averaged bed temperature was 500°C. The Base Case parameter/material properties are summarised in Table 1, noting that $k_{s}$ is the Base Case sand thermal conductivity, $M_{w}$ is the molecular weight of air and $\rho_{s}$ is the particle density of sand, which is converted to bed bulk density by the relationship presented in equation (6) (Tiskatine et al., 2017):

(6) $$\begin{array}{c} \rho_{bs}=\left(1-\phi \right)\rho_{s} \end{array}$$

**Table 1.** Base case simulation parameter set.

Parameters	Values	Units
$C_{pg}$	$-3\;\times 10^{-5}\;\left(T_{g}^{2}\right)+0.2261T_{g}+940.35\;$ ^a^	(J kg^−1^ K^−1^)
$C_{ps}$	$2.49T_{s}+39.06$ ^a^	(J kg^−1^ K^−1^)
K_g_	$-1\times 10^{-8}\left(T_{g}^{2}\right)+8\times 10^{-5}\left(T_{g}\right)+4.3\times 10^{-3}$ ^a^	(W m^−1^ K^−1^)
$k_{rad}+k_{s}$	$1.55\times 10^{-10}\left(T_{s}^{3}\right)+0.000541\left(T_{s}\right)+0.1044$	(W m^−1^ K^−1^)
$\mu_{g}$	$-9\times 10^{-12}\left(T_{g}^{2}\right)+4\times 10^{-8}\left(T_{g}\right)+6\times 10^{-6}$ ^a^	(Pa s)
$k_{i}$	$1.84\times 10^{-10}$ ^a^	(m^2^)
$P_{o}$	101,325 ^b^	(Pa)
$M_{w}$	0.029 ^b^	(kg mol^−1^)
$\rho_{s}$	2635 ^a^	(kg m^−3^)
$d_{p}$	$5.125\times 10^{-4}$ ^a^	(m)
ϕ	0.37 ^a^	(–)
$A_{sg}$	7376 ^c^	(m)
$q_{g}$	0.01 ^d^	(m s^−1^)
$T_{o}$	298.15 ^b^	(K)

a Values obtained from the study by Zanoni et al. (2017).

b Values obtained from ANSYS^®^ Student Fluent reference/default values.

c Values obtained from calculation.

d Values obtained from the study by Murray (2019).

Next, three simulations examined the influence of the initial volume-average temperature of the bed. The simulations cover the range 500°C (Base Case) to 1250°C, which represents the smouldering of a wide range of fuels/contaminants (Duchesne et al., 2020; Gerhard et al., 2020). Three simulations then analysed the influence of initial bed temperature distribution. The temperature distributions applied were Homogeneous (Figure 3(c)), Vertical Gradient (Figure 3(e)) and Horizontal Gradient (Figure 3(g)). All are approximations of observed temperature distributions in STARx beds depending on heat losses, wall insulation, fuel smouldering temperatures and other scenario-specific variables.

Three simulations then investigated the influence of varying bed bulk density from 415 to 2490 kg m^−3^; this presents a range from natural soils to operator-controlled mixtures. In addition, the influence of injection air flux was explored through three simulations in which it was incremented from 1.0 (Base Case) to 3 cm s^−1^; a range covering the equipment typically available on a STARx site. Aside from the stated variable changes, each simulation was created using the Base Case setup. This is with exception to the Homogeneous case, in which wall heat losses were disabled to prevent horizontal temperature gradients from forming during cooling.

For a cooling metric, bed-cooling time ( $t_{c}$ ) is defined as the time required for the mass-averaged temperature of the air exiting the bed ( ${\bar{T}}_{g,out}$ ) to decrease to ambient temperature. This indicates that the total heat load (i.e. the initial sensible heat storage, $E_{s}^{o}$ ) has been transferred out of the bed. Mass-averaging of the outlet air temperature was calculated at each time step by:

(7) $$\begin{array}{c} {\bar{T}}_{g,out}={\left[\frac{\sum_{i=1}^{n_{out}}T_{g,i}\rho_{g,i}\left|{\vec{v}}_{g,i}\;\cdot \;\;{\vec{A}}_{i}\right|}{\sum_{i=1}^{n_{out}}\rho_{g,i}\left|{\vec{v}}_{g,i}\;\cdot \;\;{\vec{A}}_{i}\right|}\right]}_{t} \end{array}$$

where ${\vec{v}}_{g,i}\;\cdot \;\;{\vec{A}}_{i}$ is the dot product of the cell air flow velocity and area vectors, $n_{out}$ is the total number of outlet cells and t is the instantaneous cooling time. $T_{g,i}$ and $\rho_{g,i}$ are the air temperature and density of cell i , respectively (ANSYS Inc, 2020b).

$E_{s}^{o}$ only considers sensible heat storage in the bed material as gas phase contribution is typically minimal (Alva et al., 2018; White, 2011) and assumed negligible. $E_{s}^{o}$ is calculated in the Fluent model following:

(8) $$\begin{array}{c} E_{s}^{o}=m_{s}\int_{T_{o}}^{T_{s}(t)}C_{ps}dT={\left[\left(1-\phi \right)\overset{n_{cell}}{\underset{i=1}{\sum }}h_{s,i}\rho_{s,i}V_{i}\right]}_{t=0} \end{array}$$

where $n_{cell}$ is the total number of cells in the domain, $V_{i}$ is the volume of cell i and $m_{s}$ and $C_{ps}$ are the Base Case sand mass and specific heat capacity, respectively. $h_{j,i}$ is sensible enthalpy and is calculated for either the sand or air phase as $h_{j,i}=\int_{T_{o}}^{T}C_{p,j}\left(T\right)dT$ .

The rate at which the injected air flow removes heat from the bed $\left({\dot{E}}_{outlet}\right)$ is calculated throughout each simulation as:

(9) $$\begin{array}{c} {\dot{E}}_{outlet}={\dot{m}}_{g}\int_{T_{o}}^{T_{g}(t)}C_{pg}\left(T_{g}\right)dT={\left[\overset{n_{out}}{\underset{i=1}{\sum }}h_{g,i}\rho_{g,i}{\vec{v}}_{g,i}\;\cdot \;\;{\vec{A}}_{i}\right]}_{t} \end{array}$$

where ${\dot{m}}_{g}$ and $C_{pg}$ are the outlet mass flow rate and specific heat capacity of air, respectively. Important to note is that ${\vec{v}}_{g,i}$ is directly influenced by the applied injection air flux. Heat is also removed from the bed through the wall boundary by lateral heat transfer (i.e. conduction). The wall heat loss rate from the sand phase is calculated as:

(10) $$\begin{array}{c} {\dot{E}}_{wall}={\left[\overset{n_{wall}}{\underset{i=1}{\sum }}\frac{k_{s}}{\Delta n_{wall}}\left(T_{w}-T_{s}\right)\right]}_{t} \end{array}$$

where ∆ $n_{wall}$ is the solid cell to wall surface distance, $n_{wall}$ is the total number of near-wall solid cells and $T_{w}$ is the wall surface temperature (ANSYS Inc, 2020a).

Total outlet and wall heat losses are calculated by integrating equations (9) and (10) over the cooling durations. The average heat loss rate through either boundary was determined by dividing total heat loss through that boundary by the bed-cooling time.

In addition, for each simulation, the distribution of air flow leaving the top of the bed was assessed. Figure 3 displays that four ‘sampling locations’ were arranged along the outlet boundary of the domain. Each sampling location was created as a line surface, 1 m in length, and equally spaced across the outlet. Mass flow data were integrated over the cooling duration to determine the total percent of air flow (by mass) through each location.

## Results and discussion

### Base Case

The Base Case simulation resulted in a cooling time of 7.7 days. The majority of heat loss was through the bed outlet via the exiting hot air flow (~ 99%), while wall heat losses were negligible (~ 1%). This corresponded to average heat loss rates of approximately 40 kW and 0.3 kW, respectively. Initial sensible heat storage in the bed (i.e. the total heat load) was 7364 kWh_th_.

**Figure 4.**
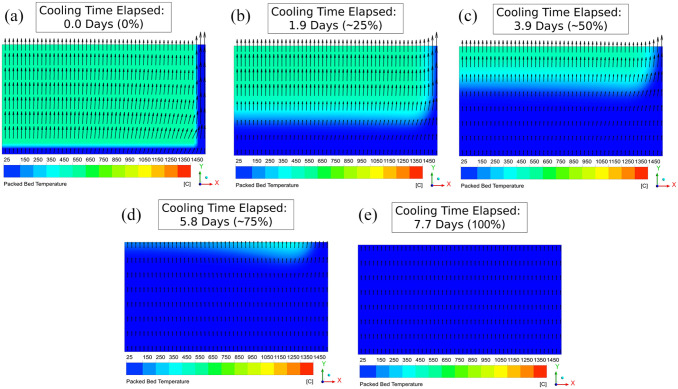
Bed temperature distributions observed throughout the Base Case simulation: (a) at the start of cooling, (b) after 25% of total cooling time was completed (1.9 days), (c) 50% (3.9 days), (d) 75% (5.8 days) and (e) at the end of cooling (7.7 days).

Figure 4 displays the evolution of bed temperature distribution with time. In particular, a ‘heat bulb’ was observed to deflect air flow towards the near-wall region, where flow velocities were greatest (~ 11 cm s^−1^ maximum velocity, compared to ~ 8 cm s^−1^ closer to the bed centre). This thermally induced air channelling occurred due to spatial and temporal variations in pneumatic conductivity ( $K_{p}$ ). $K_{p}$ refers to the ease at which air can flow through a porous bed (Zanoni et al., 2021):

(11) $$\begin{array}{c} K_{p}=\frac{k_{i}\rho_{g}g}{\mu_{g}} \end{array}$$

where $\rho_{g}$ is the air density and $\mu_{g}$ is the air viscosity, $k_{i}$ is the intrinsic permeability of the bed and g is gravitational acceleration. As $\rho_{g}$ decreases and m $\mu_{g}$ increases with increasing temperature, $K_{p}$ decreases. Therefore, higher temperature regions of the bed are less conductive to air flow. This effect is similar to how physically induced air channelling can occur due to permeability heterogeneity (Rashwan et al., 2021c; Wang et al., 2021) and porosity variations (Dalman et al., 1986; Laguerre et al., 2006; Meier et al., 1991).

Recall that exit airflow sampling locations (A through D) were included (Figure 3(a)). For the Base Case, this revealed that near-wall air flow (through sampling location D) accounted for the greatest portion (~ 29%) of the total air flow (Figure 6). Preferential air flow in this region led to the adjacent region (sampling location C) experiencing the least proportion of air flow (~ 23%). This is because the C sampling location was positioned overtop of the ‘low $K_{p}$ heat bulb’ (Figure 4(d)). Following the sharp flow reduction at location C, air flow uniformity increased with greater distance from the wall, towards the bed centre (sampling locations A and B).

Comparison of Figure 4(d) and (e) reveals that ~ 2 days was required for complete cooling of the ‘heat bulb’. Overall, this highlighted how air divergence can be problematic for bed cooling. Air flow will tend to bypass higher temperature regions in favour of cooler ones, prolonging the cooling process. Of particular concern is bypassing through regions near containment walls, where heat losses lead to lower temperatures and higher $K_{p}$ . This phenomenon is known but has not been studied and is much further examined – particularly with respect to impact on cooling times – in the upcoming sections. Note that for all simulations, video files detailing the evolution of bed temperature distribution are provided in the Supplemental Material.

### Initial volume-averaged temperature

**Figure 5.**
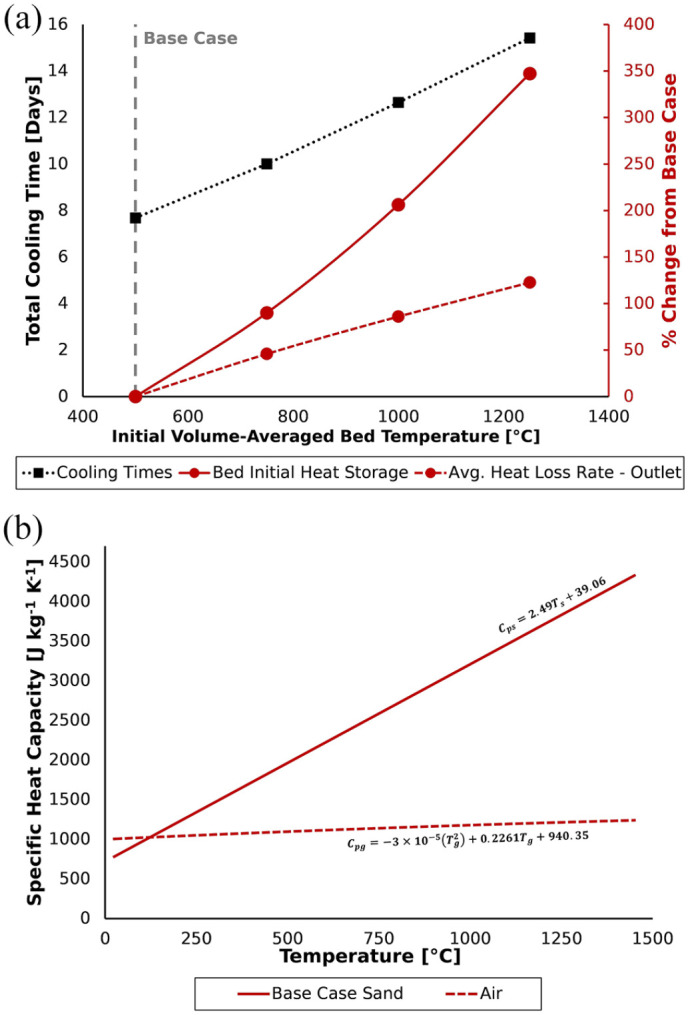
(a) Total cooling times and the percentage change (relative to the Base Case simulation) of initial heat storage in the bed (i.e. the heat load) and the average outlet heat loss rate with variation of initial volume-averaged bed temperature. (b) Comparison of Base Case sand and air specific heat capacities over the range of bed temperatures implemented.

Increased initial volume-averaged bed temperature led to a nearly linear increase in cooling time (Figure 5(a)) from 7.7 days in the Base Case (500°C) to 15.4 days in the 1250°C case. This was primarily due to the greater increase of the bed total heat load with temperature in comparison to the average outlet heat loss rate (Figure 5(a)). Acknowledging that the mass of the bed and the mass flow rate of air were held constant between these simulations, heat load and outlet heat loss changes were solely dependent on the specific heat capacities of the Base Case sand and air, respectively.

The increase of heat storage in packed beds due to the increase of bed material specific heat capacity with temperature is well known (Aly and El-Sharkawy, 1990; Ammar and Ghoneim, 1991; Elouali et al., 2019; Hrifech et al., 2020; Kumar and Shukla, 2015; Tiskatine et al., 2017). In agreement, Figure 5(b) confirms increase of the Base Case sand specific heat capacity with temperature. Notice that this increase is more rapid than that of the specific heat capacity of air. This reflects the previous comparison made between the increases of heat load and the average outlet heat loss rate as bed temperature was increased. Therefore, in addition to greater total heat loads at higher temperatures, the cooling process was also inhibited by the air’s specific heat capacity. This effectively limited the rate at which heat could be transferred to the air and removed from the bed. These results call attention to higher temperature STARx applications (e.g. > 900°C for PFAS treatment), which are intrinsically susceptible to longer cooling times.

### Initial temperature distribution

**Figure 6.**
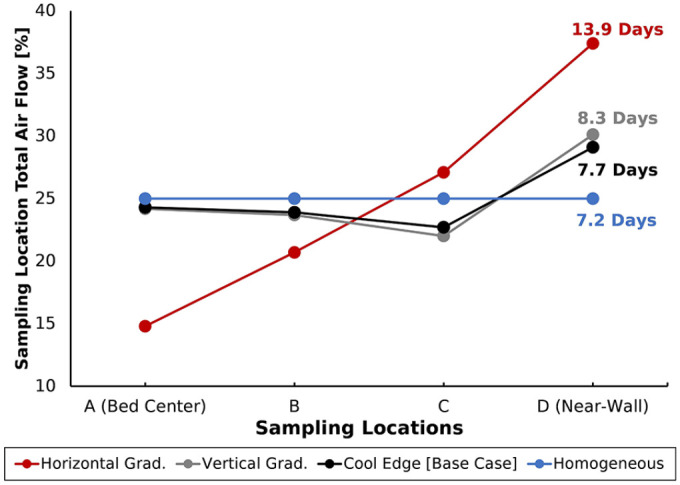
Total air flow distribution among the outlet sampling locations for each initial temperature distribution. Corresponding cooling times are labelled.

Varying the initial temperature distribution investigated how spatial temperature inhomogeneities altered air flow distribution in the bed and impacted cooling time. For the Homogeneous distribution, bed $K_{p}$ homogeneity was featured at cooling start (Figure 3(d)). The even distribution of total air flow (Figure 6) indicates that this temperature distribution prevented any air divergence from occurring. As a result, the Homogeneous distribution led to the fastest cooling time, 7.2 days, a 0.5 day (or 6%) reduction from the Base Case.

In contrast, the Vertical and Horizontal Gradient cases featured temperature variation in the axial and lateral directions, respectively (Figure 3). Note that the Horizontal Gradient featured high $K_{p}$ ‘layers’ aligned parallel to the primary flow direction (inlet to outlet). Conversely, the majority of temperature layers in the Vertical Gradient were normal to the primary flow direction, with only the cool near-wall region extending parallel to it. As such, Figure 6 shows that total air flow distribution varied drastically across the bed for the Horizontal Gradient and led to the longest cooling duration (13.9 days, Figure 6). By measure, the total air flow distribution of the Vertical Gradient resembled that of the Base Case, with air divergence only near to the wall. Accordingly, the Vertical Gradient cooling time (8.3 days, Figure 6) was an increase of only 0.6 days (or 8%) from the Base Case. Importantly, these observations make evident that the degree of air divergence and resulting cooling time increases are driven by the extent of temperature variation perpendicular to the dominant air flow directions in the bed.

Bed temperature inhomogeneity has been investigated relative to its role in smouldering reaction dynamics. Namely, its influence on injected air flow rates (Levin and Lutsenko, 2006, 2008), non-uniform air flux (Rashwan et al., 2021c), pressure changes within smouldering reactors (Zanoni et al., 2021) and reaction front instability (Chen et al., 2018; Rabinovich et al., 2016). Relatedly, the results of this work provide the first investigation into how bed temperature inhomogeneities derived during the smouldering phase can have ramifications for the cooling phase. Specifically, by resolving the flow field within the bed (Supplement D) and highlighting that the occurrence of thermally induced air flow redistribution leads to longer cooling times.

### Porous bed bulk density

**Figure 7.**
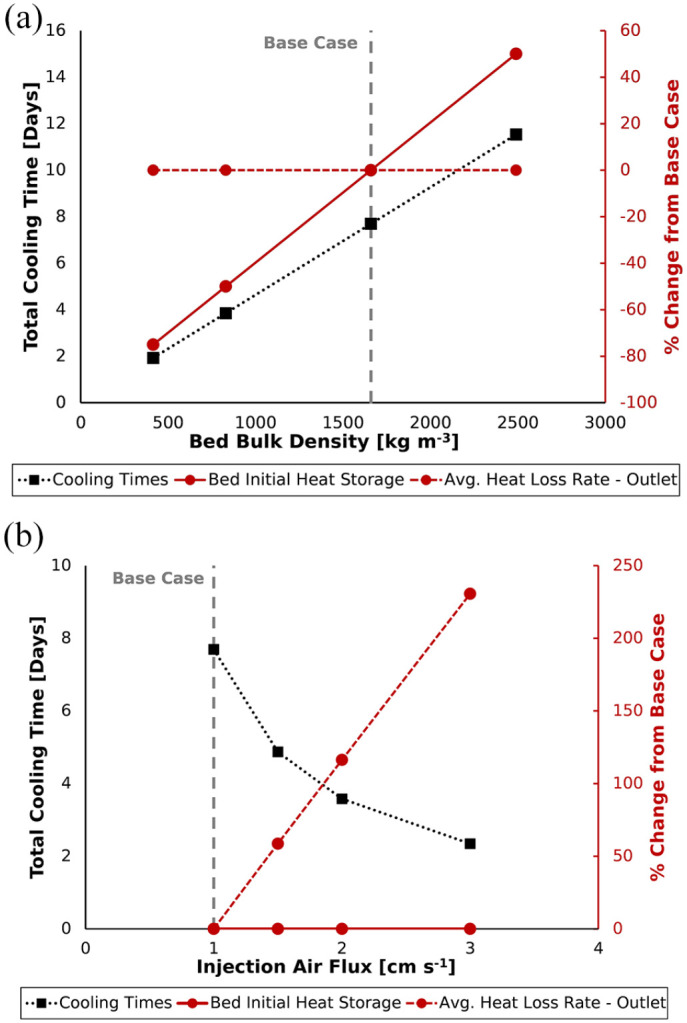
Total cooling times and the percentage change (relative to the Base Case simulation) of initial heat storage in the bed and the average outlet heat loss rate due to variation of (a) bed bulk density and (b) applied injection air flux.

For liquid waste smouldering, the bed bulk density can be controlled by changing the type of porous matrix material chosen during system set-up. Recalling equation (6), it is seen that the bulk density of a material is a function of its particle density $\left(\rho_{s}\right)$ as well as its porosity (ϕ) . Relatedly, it has been reported that the heat load in a packed bed (relative to a set temperature) is increased through use of materials with greater particle density (Aly and El-Sharkawy, 1990; Elouali et al., 2019; Hrifech et al., 2020; Romaní et al., 2019) and/or reduced porosity (Abdel-Salam et al., 1991; Ismail and Stuginsky, 1999). Altering either of these parameters in this manner would also lead to an increase in the bed bulk density (equation (6)). In agreement, Figure 7(a) displays that direct increases of the bed bulk density caused a linear increase of total heat load.

It was also seen that the average outlet heat loss rate was unaffected by bulk density variations (Figure 7(a)). Rather, it remained unchanged as all beds were initialised at Base Case temperature and distribution (500°C, Cool Edge). This meant that sand–air heat transfer was driven by the same temperature differentials regardless of changes to bed bulk density. Therefore, with average heat removal rate constant and only the heat load varied between each simulation, cooling time was observed to be linearly proportional to the bed bulk density (Figure 7(a)). Altogether, this indicated that porous matrix material selection (a key step of system design) offers STARx operators substantial cooling time control.

### Injection air flux

Air flux increases were found to reduce cooling time significantly (Figure 7(b)), as greater injection air fluxes enhanced the sand-to-air heat transfer process. This is inferable as the sand–air heat transfer coefficient $\left(h_{sg}\right)$ is a function of Reynolds number (equation (5)), which increases at higher flow velocities. Significant increase of outlet heat loss rates were indicative of heat transfer enhancement and faster heat load removal. For instance, the 3 cm s^−1^ air flux increased the average outlet heat loss rate by 231% from the Base Case (Figure 7(b)) and reduced cooling times to 2.3 days. This was a 70% decrease from the 7.7 day Base Case cooling time. Increasing instead to only 2 cm s^−1^ reduced cooling time by 53% relative to the Base Case (from 7.7 days to 3.6 days) and increasing only to 1.5 cm s^−1^ resulted in a 36% reduction (from 7.7 days to 4.9 days). These results agree qualitatively to research on thermal energy systems, which indicated that increased heat transfer fluid injection rates reduced thermal discharging times (Gautam and Saini, 2020; Kim et al., 2017; Lugolole et al., 2019; Niyas et al., 2015; Suresh and Saini, 2020; Xu et al., 2012). The cooling time reduction here was revealed to be non-linear with air flux (Figure 7(b)).

### Optimisations for cooling time reduction

This work reveals new optimisation opportunities for STARx systems with respect to cooling time reduction:

1. Increased injection air flux during the cooling phase. Greater injection air fluxes enhanced sand–air heat transfer and led to significant increases of outlet heat loss rate. All air flux increases beyond the Base Case (1 cm s^−1^) led to reduced cooling times. This highlighted a strong, system-embedded approach to cooling duration control for site engineers as air injection equipment is an essential operational component of STARx and other thermal waste treatment systems.
2. The use of packed bed materials with lower bulk densities. This is supported by the linear proportionality observed between cooling time and bulk density. Importantly, bulk density can be controlled by the operator during system set-up. Examples of potential alternative inert porous matrix materials to quartz sand include alumina spheres and pumice, among many others. However, aside from cooling time reductions, alternative bed materials should also be cost effective and commonly available (Savron Solutions, personal communication). Both of which are advantages that sand offers when used in packed bed smouldering (Yermán et al., 2015) and thermal energy storage (Alva et al., 2018) systems alike.
3. Enhancement of containment wall insulation. Horizontal temperature gradients were found to influence substantial air divergence and increase cooling time. In agreement, temperature homogeneity led to reduced cooling times. Greater homogeneity can be influenced by minimising lateral heat losses via improved wall insulation, combating the negative influence of thermally induced air channelling on cooling times, particularly, at increased bed temperatures and in the near-wall region where air divergences effects are more pronounced.
4. Although not studied here, this work suggests that an additional option for cooling time reductions would be the use of alternative heat transfer fluids – with specific heat capacities greater than that of air – during the cooling phase. Higher specific heat capacities would facilitate increased sand–air heat transfer and outlet heat loss rates, for faster heat load removal. Examples include thermal oils, as used in some PBTES systems (Alva et al., 2018; Gautam and Saini, 2020). With that said, air is generally favourable for porous bed cooling due to its chemical stability, non-toxicity, non-flammability as well as unlimited and effectively cost-free availability (Gautam and Saini, 2020; Park et al., 2014).

It is noted that air flux (Pironi et al., 2009, 2011; Rashwan et al., 2016; Zanoni et al., 2019a, 2019b), bed bulk density (Hu et al., 2020; Huang and Rein, 2017; Krause et al., 2006; Prat-Guitart et al., 2016) and wall heat loss/insulation (Lin and Huang, 2021; Rashwan et al., 2021c, 2021d; Yermán, 2016) have all been considered with respect to their influence on the smouldering reaction. However, the new cooling optimisations reveal how key parameters quantitatively influence the overall efficiency and effectiveness of commercial smouldering systems beyond the smouldering phase. Therefore, total system assessment is imperative ahead of real-world implementation of these optimisations. This includes properly accounting for the foreseeable economic and implementation complexities (e.g. equipment, material adjustments) of these optimisations.

## Conclusion

This research explored the influence of key system parameters on the cooling time of a waste thermal treatment bed. This investigation led to improved understanding of bed-cooling dynamics and the identification of optimisations for cooling time reduction. In addition to applied smouldering systems, the processes and optimisations detailed in this research are highly applicable to a wide range of engineering applications which involve air flow through hot beds and solid particle cooling. The main findings of this research are summarised as follows:

- A near linear relationship was found between decreased cooling time and the decrease of both the volume-averaged bed temperature and bed bulk density;
- Bed temperature variation perpendicular to the dominant air flow direction caused undesirable air divergence effects, particularly in the near-wall region, and elongated cooling times;
- A non-linear relationship between increased injection air flux and reduced cooling times was revealed. Modest increases in air flux led to significant cooling time reductions;
- Based on these results, increasing injection air fluxes, use of lower bulk density bed materials and improving containment wall insulation were identified as practical system optimisations to reduce bed-cooling times.

## Supplemental Material

sj-docx-1-wmr-10.1177_0734242X221076308 – Supplemental material for Understanding, controlling and optimising the cooling of waste thermal treatment beds including STARx Hottpads Click here for additional data file. Supplemental material, sj-docx-1-wmr-10.1177_0734242X221076308 for Understanding, controlling and optimising the cooling of waste thermal treatment beds including STARx Hottpads by Ryan B Morales, Christopher T DeGroot, Grant C Scholes and Jason I Gerhard in Waste Management & Research

sj-docx-2-wmr-10.1177_0734242X221076308 – Supplemental material for Understanding, controlling and optimising the cooling of waste thermal treatment beds including STARx Hottpads Click here for additional data file. Supplemental material, sj-docx-2-wmr-10.1177_0734242X221076308 for Understanding, controlling and optimising the cooling of waste thermal treatment beds including STARx Hottpads by Ryan B Morales, Christopher T DeGroot, Grant C Scholes and Jason I Gerhard in Waste Management & Research
