# Supplementary material for: Understanding, controlling and optimising the cooling of waste thermal treatment beds including STARx Hottpads
Source: Waste Manag Res. 2022 Mar 21;40(9):1390–401. doi: 10.1177/0734242X221076308 (PMC9393652; doi:10.1177/0734242X221076308)
Supplement: sj-docx-1-wmr-10.1177_0734242X221076308 – Supplemental material for Understanding, controlling and optimising the cooling of waste thermal treatment beds including STARx Hottpads [file sj-docx-1-wmr-10.1177_0734242X221076308.docx]

Understanding, Controlling and Optimizing the Cooling of Waste Thermal Treatment Beds including STARx Hottpads

- Supplemental Material –

Ryan B Morales^[[1]](#footnote-1)^, Christopher T DeGroot^2^, Grant Scholes^3^, and Jason I Gerhard^1,^4

Submitted to Waste Management & Research

# Supplement A: Additional Model Setup Information

The 2D domain used for all simulations is seen below in Figure A1. The boundary conditions for the spatially coincident gas and solid phases are also indicated in the figure. Table A1 presents a summary of all applied boundary conditions and their positions.

| 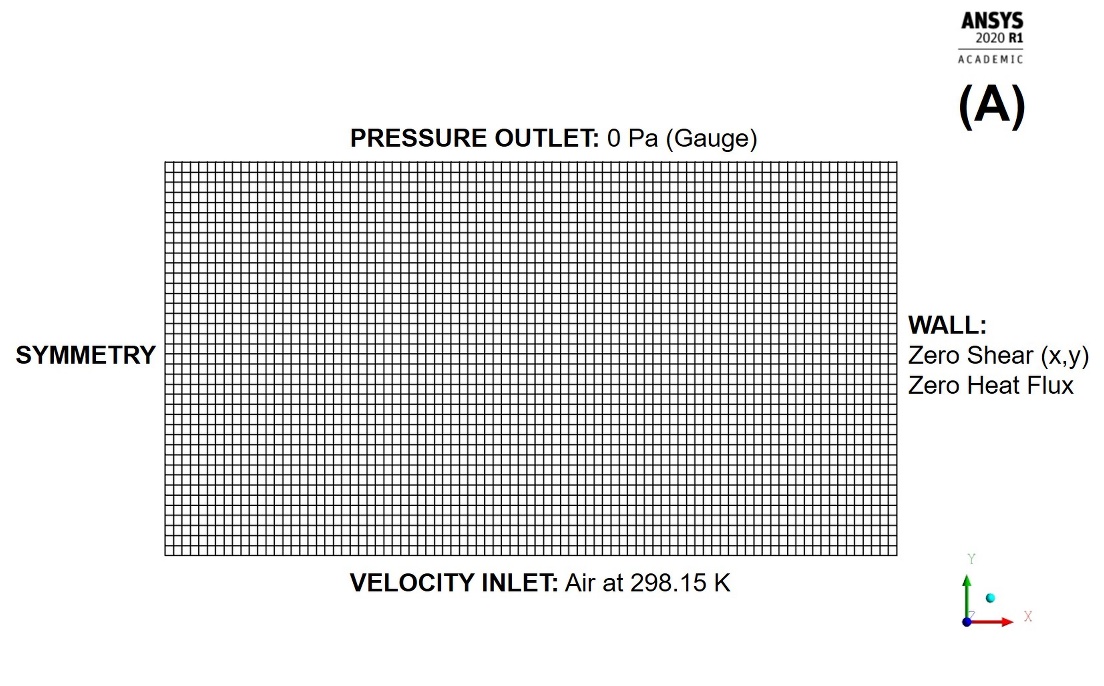 | 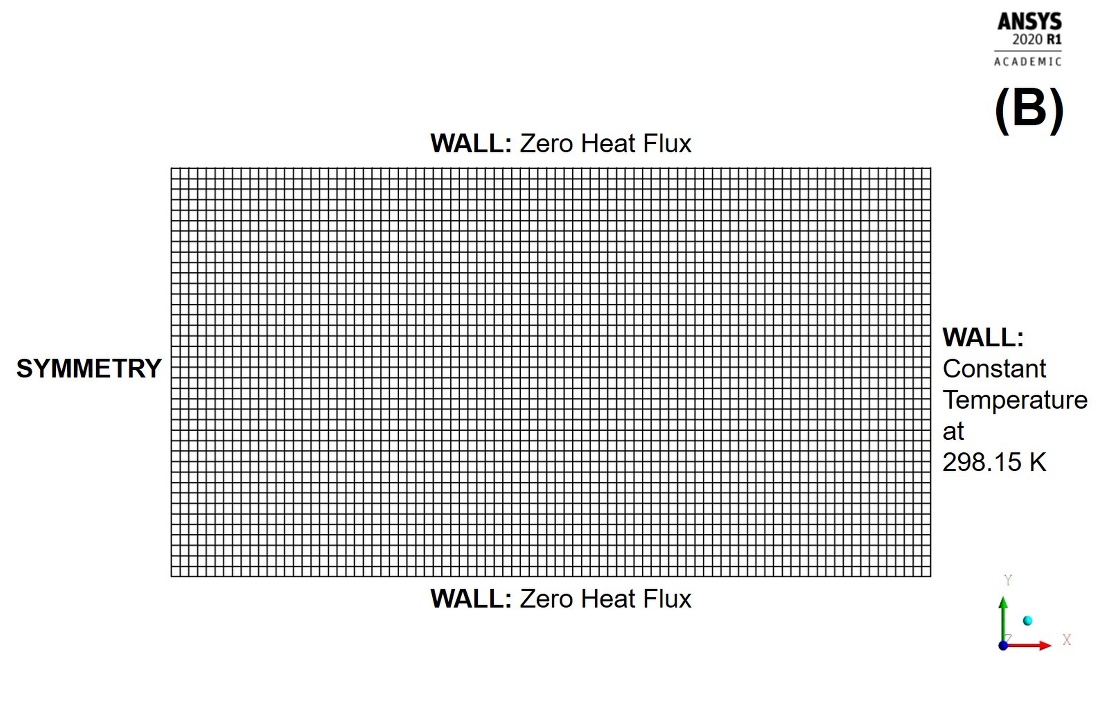 |
| --- | --- |
| 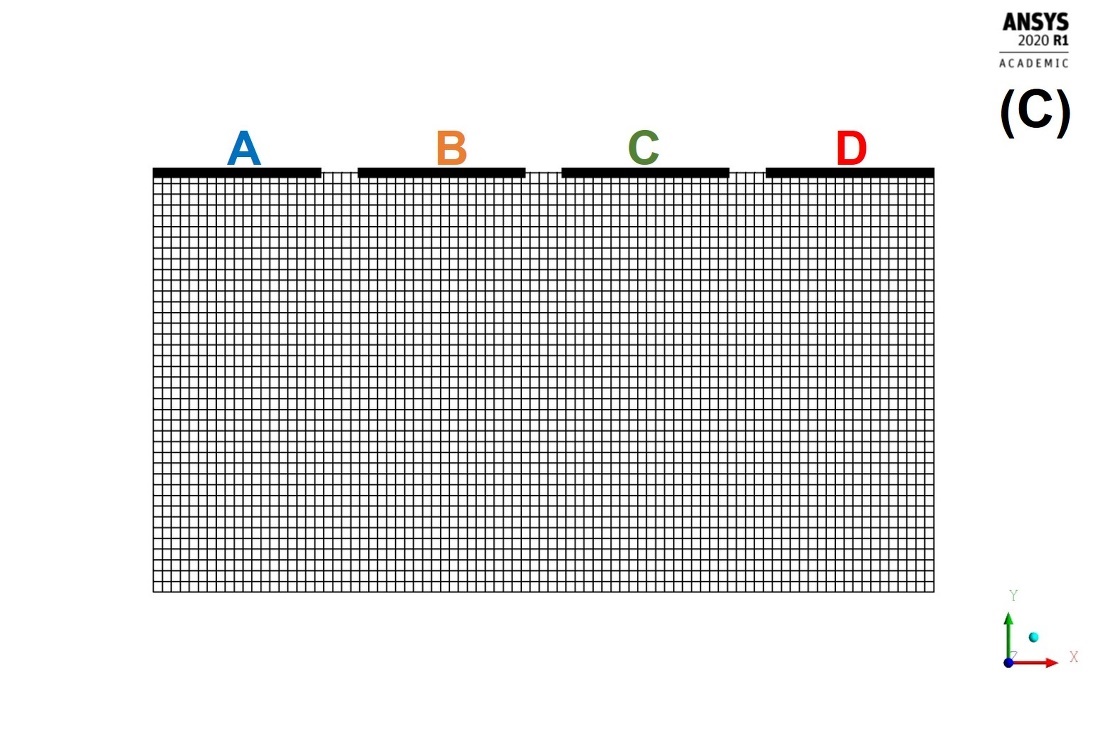 | |

Figure A1: Visualization of the two-dimensional domain geometry and arrangement of cells. Figure (A) and (B) indicate the boundary conditions applied to the gas and solid phases, respectively. Figure (C) presents the positioning of air mass flow rate “sampling locations” across the outlet boundary.

Figure A1 also displays the positioning of the “sampling locations” across the top (outlet) boundary of the domain. The sampling locations were constructed as 1m line surfaces. Data points generated by Fluent on these line surfaces consider nearby cell values for the computation the mass flow rate of air through each sampling location (ANSYS Inc., 2020).

Table A1: Summary of Base Case Boundary Conditions

| Boundary  Type | Position | Time Interval | Momentum Conditions | | Thermal Conditions | |
| --- | --- | --- | --- | --- | --- | --- |
|  |  |  | Gas Phase | Solid Phase | Gas Phase | Solid Phase |
| Velocity  Inlet | $y = 0 m$  $0 m\leq x\leq4.65 m$ | $t_{o}\to t_{c}$ | 0.01 m s^-1^ | - | 25$^{\circ}$C | Zero heat flux |
| Wall | $0 m\leq y\leq2.5 m$  $x=4.65 m$ | $t_{o}\to t_{c}$ | Stationary wall  X,Y zero shear | - | Zero Heat Flux | 25$^{\circ}$C |
| Symmetry | $0 m\leq y\leq2.5 m$  $x=0 m$ | $t_{o}\to t_{c}$ | - | - | - | - |
| Pressure Outlet | $y = 2.5 m$  $0 m\leq x\leq4.65 m$ | $t_{o}\to t_{c}$ | 0 Pa (Gauge) | - | 25$^{\circ}$C (Backflow) | Zero heat flux |

Fluent solver settings for all simulations were chosen based on best practices indicated in the ANSYS*^®^* Fluent User’s Guide 2020 R1/R2 (ANSYS Inc., 2020) and to enhance solution accuracy and decreasing total computational time. Accuracy was prioritized using second order spatial and temporal discretization methods and also sufficiently low residual thresholds (1x10^-5^ for mass and momentum equations, 1x10^-6^ for energy equations). A large Flow Courant Number (10^7^) was selected to decrease computational time. This value was applied based on recommendation from (ANSYS Inc., 2020) and the unconditional numerical stability of implicit solvers. The Flow Courant Number refers to the Fluent’s method of implicit relaxation for its pressure-based solver. Larger Flow Courant Numbers accelerate the solution while smaller numbers improve stability (ANSYS Inc., 2020). Solution acceleration was prioritized because of the high-level of grid uniformity, sufficiently low error due to spatial and temporal discretization (see Supplement B and C), and laminar flow condition (due to small Darcy fluxes).

# Supplement B: Model Validation Details

Temperature data from a smouldering experiment as part of Zanoni et al. (2021) was used for model validation. The experiment was conducted in a column reactor (0.8 cm stainless-steel walls) with a 73 cm packed bed and 5.4 cm radius. The reactor was wrapped with a 4.1 cm thick thermal insulating foam blanket and enclosed by a 0.1 cm thick aluminum jacket. During the experiment, heat and air injection were applied at the base of the column to ignite a forward propagating smouldering front. Heat was supplied by a 500 W external radiative cone heater, at a stabilized temperature of 1000 $^{\circ}$C. An air flux of 0.05 m s^-1^ was applied via a mass flow-controlled compressed air supply. Granular activated carbon (GAC) was mixed with sand ($d_{p}$ = 0.88 mm) to form the fuel bed within the column. The bed was instrumented with 25 thermocouples equally spaced along the reactor centreline (i.e., 0.03 m apart starting at 0.01 m above the column base, Figure B1). Two sets of radially situated thermocouples (each set consisting of 0 cm [centerline], 1.5 cm, 2.8 cm, 4.1 cm, and 5.4 cm [wall] thermocouple positions) were also placed at reactor heights of 25 cm and 49 cm.


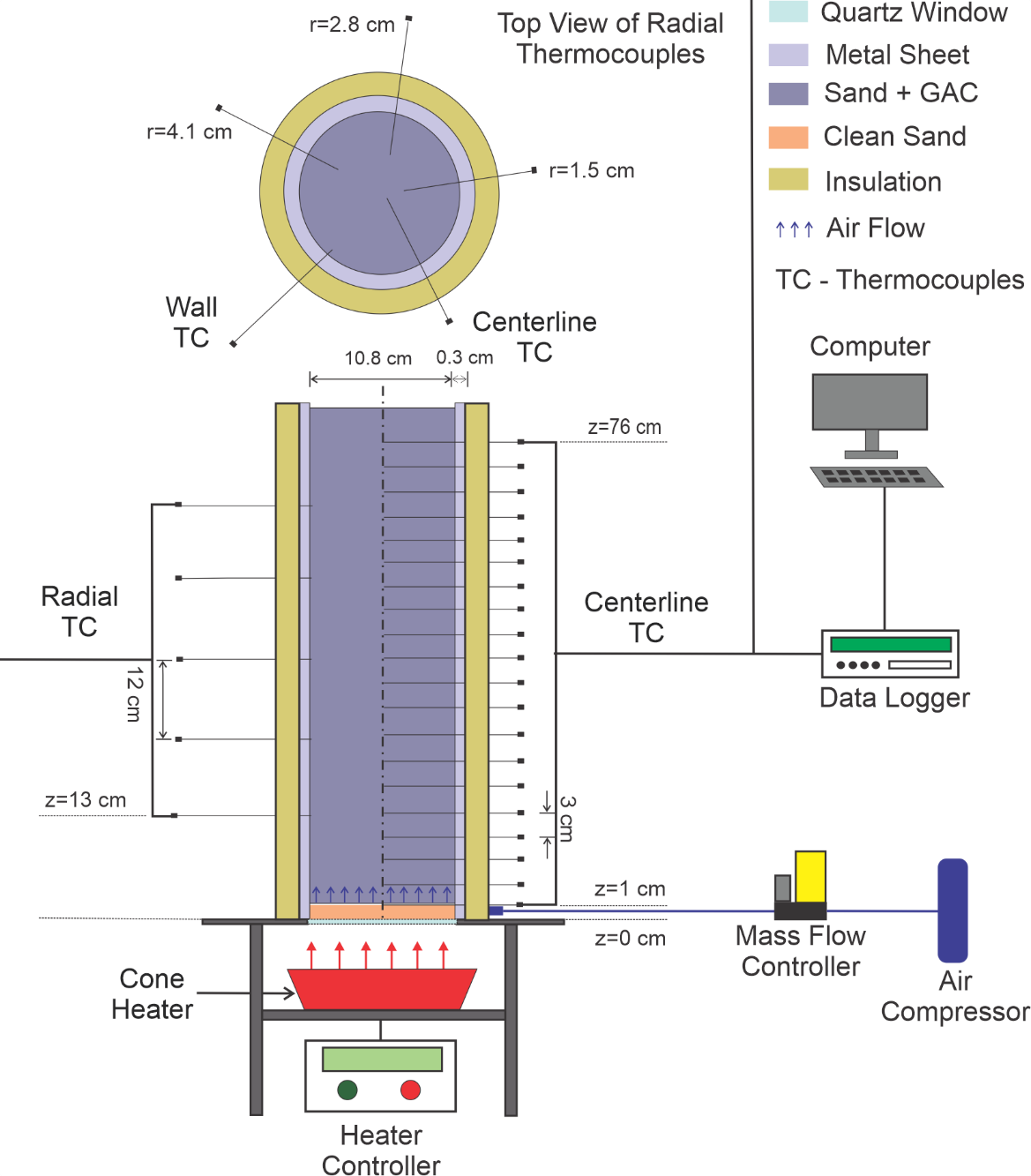


Figure B1: Visualization of the column smouldering experiment referenced for model validation. Adapted from Zanoni et al. (2021).

As this study is focused on the cooling phase after smouldering treatment, only the temperature data from the cooling phase of the smouldering experiment was used for validation. Figure B2 shows that smouldering completed after 16430 s, which was identified by the peak centerline temperature (678 °C) at the top of the column (70cm)**.** Because applied smouldering is often fuel-limited, the peak temperature signals complete smouldering (i.e., when all fuel is burned away). Therefore, the bed during the cooling phase was assumed to be free of fuel (i.e., only hot clean sand), which is commonly observed in many applied smouldering studies (Baud et al., 2015; Martins et al., 2010; Pironi et al., 2011).


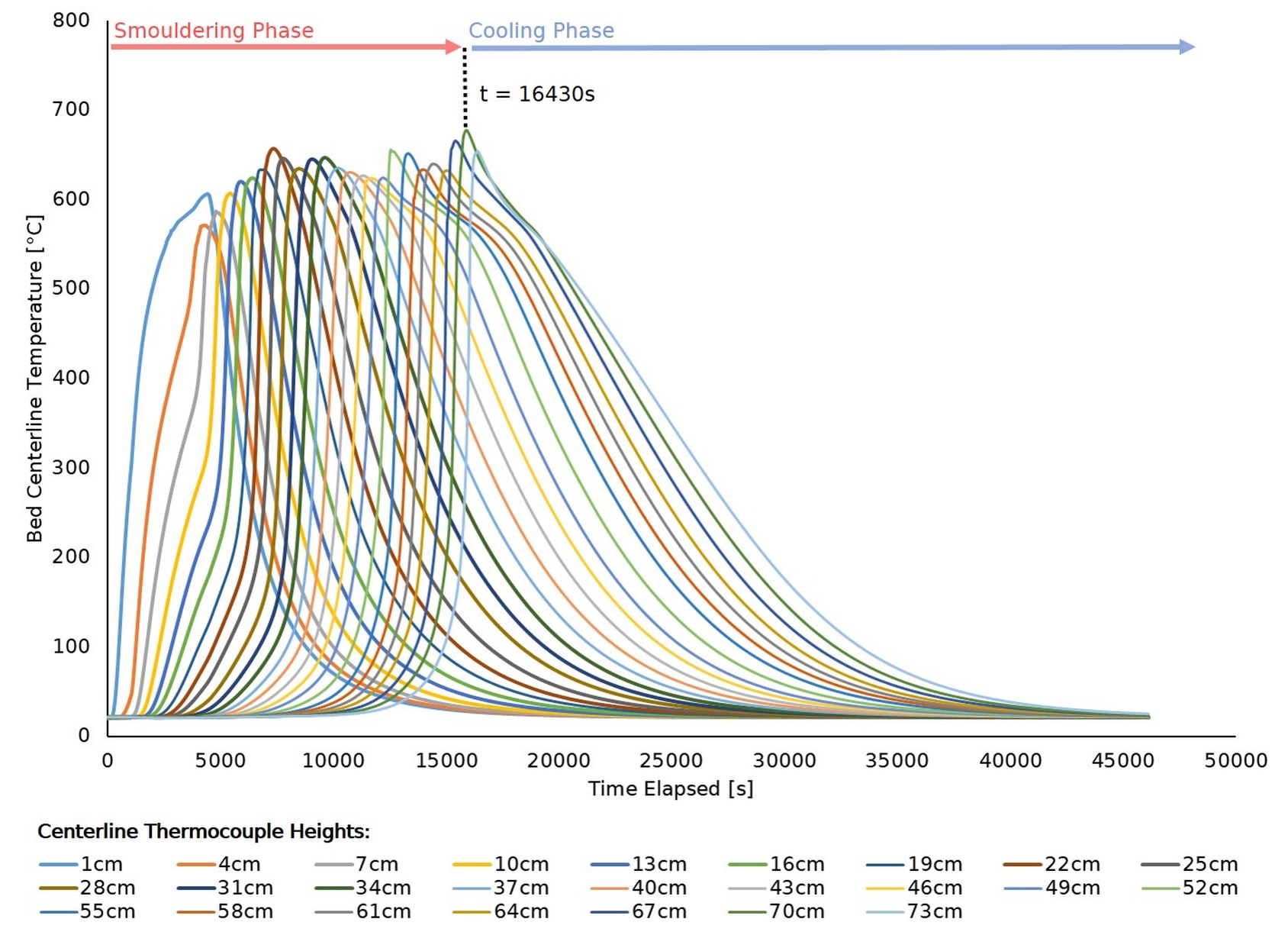


Figure B2: Transient centerline temperatures observed during the column smouldering experiment. The start of cooling/end of smouldering is identified at t = 16430s.

To simulate the cooling phase, a 2D axis-symmetric domain was created to represent this experimental setup. Both the bed and wall were included in the domain; however, the foam-alumina jacked was not modelled; instead, the radial heat loss coefficient ($H$) from Zanoni et al. (2021) was applied at the outer boundary of the wall (Figure B3). Its value is presented in the Table B1, which details all parameters used in the model validation simulation.


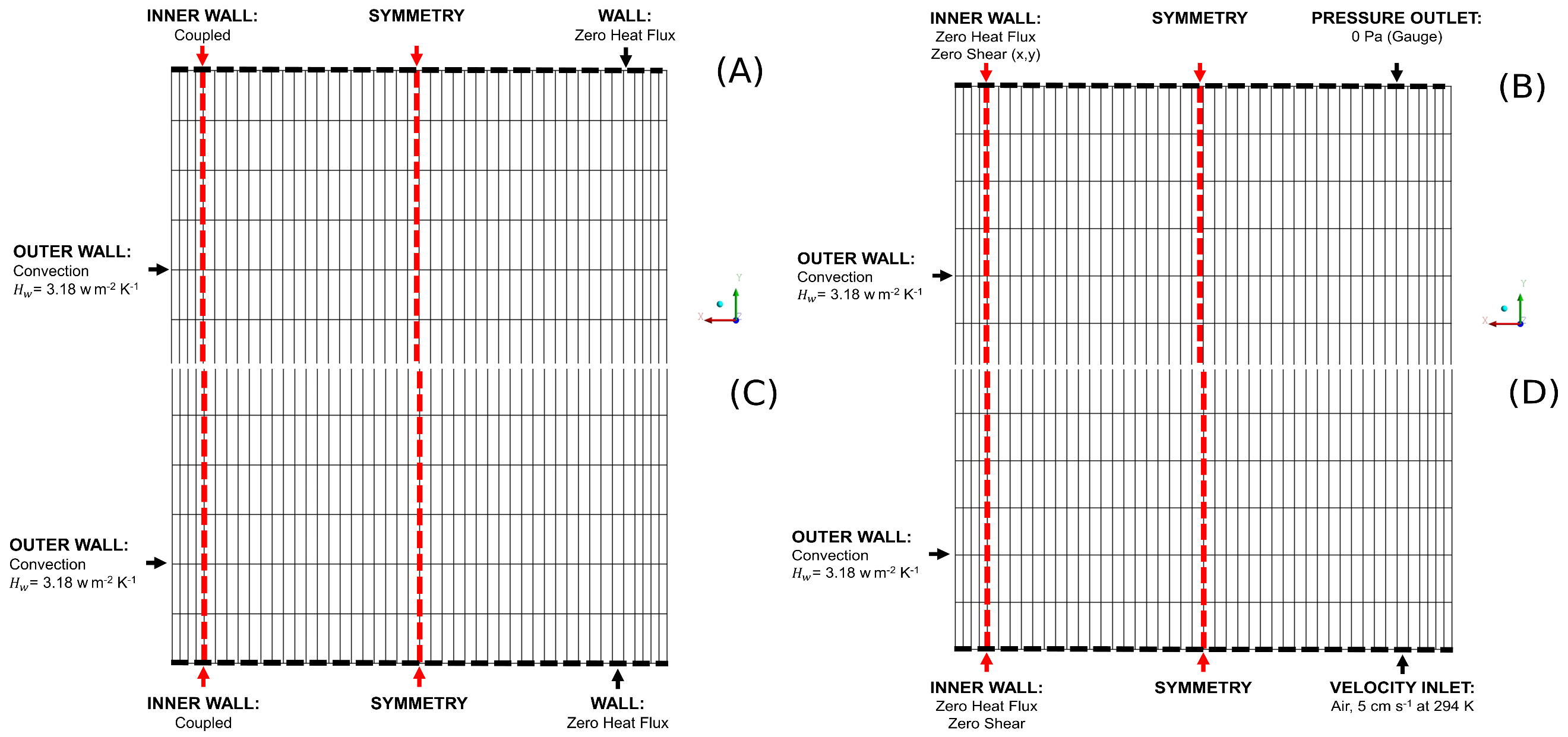


Figure B3: Boundary conditions applied for the solid (A and C) and gas (B and D) phases.

Table B1: Summary of Parameters Used for the Model Validation Simulation

| **Parameter** | **Value** | **Unit** | **Reference** |
| --- | --- | --- | --- |
| $C_{pg}$ | $-3 \times{10}^{-5} \left( T_{g}^{2} \right)+0.2261T_{g}+940.35$ | $J kg^{-1}K^{-1}$ | (Zanoni et al., 2017) |
| $C_{ps}$ | $1.75T_{s}+340.32$ | $J kg^{-1}K^{-1}$ | (Zanoni et al., 2017) |
| $C_{p,steel}$ | $440.8$ | $J kg^{-1}K^{-1}$ | (Miry, n.d.) |
| $k_{g}$ | $-1\times{10}^{-8}\left( T_{g}^{2} \right)+8\times{10}^{-5}\left( T_{g} \right)+4.3\times{10}^{-3}$ | $W m^{-1}K^{-1}$ | (Zanoni et al., 2017) |
| $k_{rad}+k_{s}$ | $1.55\times{10}^{-10}\left( T_{s}^{3} \right)+0.000541\left( T_{s} \right)+0.1044$ | $W m^{-1}K^{-1}$ | (Zanoni et al., 2017) |
| $k_{steel}$ | $z-direction = 14.76$  $r-direction = 5.96$ | $W m^{-1}K^{-1}$ | (Miry, n.d.) |
| $\mu_{g}$ | $-9\times{10}^{-12}\left( T_{g}^{2} \right)+4\times{10}^{-8}\left( T_{g} \right)+6\times{10}^{-6}$ | $Pa s$ | (Zanoni et al., 2017) |
| $k_{p}$ | $2.54\times{10}^{-10}$ | $m^{2}$ | (Zanoni et al., 2017) |
| $P_{o}$ | $101,325$ | $Pa$ | Fluent Default |
| $M_{w}$ | $0.02897$ | $kg mol^{-1}$ | Fluent Default |
| $\rho_{s}$ | $2650$ | $kg m^{-3}$ | (Zanoni et al., 2017) |
| $\rho_{steel}$ | $8000$ | $kg m^{-3}$ | (Miry, n.d.) |
| $d_{p}$ | $8.80\times{10}^{-4}$ | $m$ | (Miry, n.d.) |
| $\phi$ | $0.4$ | - | (Zanoni et al., 2017) |
| ${A_{s,sp}}/{V_{sp}}$ | $4091$ | $m^{-1}$ | Calculated |
| $q_{g}$ | $0.05$ | $m s^{-1}$ | (Miry, n.d.) |
| $H_{w}$ | $3.18$ | $W m^{-2} K^{-1}$ | (Miry, n.d.) |
| $T_{amb}$ | $294$ | $K$ | (Miry, n.d.) |

Due to the dual cell approach of Fluent’s Non-Equilibrium Thermal Model (LTNE), the solid and gas phases both required boundary conditions. For the solid phase, Figure B3A and Figure B3C provide a visual summary of the applied boundary conditions. The inner wall of the reactor was set as a coupled wall to allow for conductive heat transfer between the bed and the wall. The inlet (bottom boundary) and outlet (top boundary) of the reactor were set as zero heat flux walls as: 1) no heat flux was applied to the solid zone at the inlet during cooling and 2) heat was predominantly transferred by convection through the outlet boundary via the gas phase. Figure B3B and Figure B3D summarize the boundary conditions applied to the gas phase. The inner wall boundary was set as a zero shear and zero heat flux boundary. The zero-shear condition was reflective of the low $Re$ flow (i.e., negligible velocity boundary layer effects). The zero-heat flux condition was due to assuming negligible conductive heat transfer from the gas phase to the wall. A velocity inlet boundary was applied at the bottom surface of the domain to allow for air injection at 5 cm s^-1^ and 21 °C. A pressure outlet boundary was applied at the top surface and set to 0 Pa (gauge) as air exits the reactor to an overhead “hood” space, which is at ambient pressure. Finally, the symmetry axis boundary condition was common to both phases and was positioned along the centerline of the domain (Figure B3). The outer wall boundary condition was applied to the portion of the domain representative of the steel reactor wall (i.e., the left edge of the domain in Figure B3). The main boundary conditions and overall computational domain are presented in Figure B4. Furthermore, all boundary conditions are summarized in Table B2. In general, this model formulation followed the same methodology described in the *Methods* section of the manuscript but used parameters specific to this validation experiment and 2D governing equations.


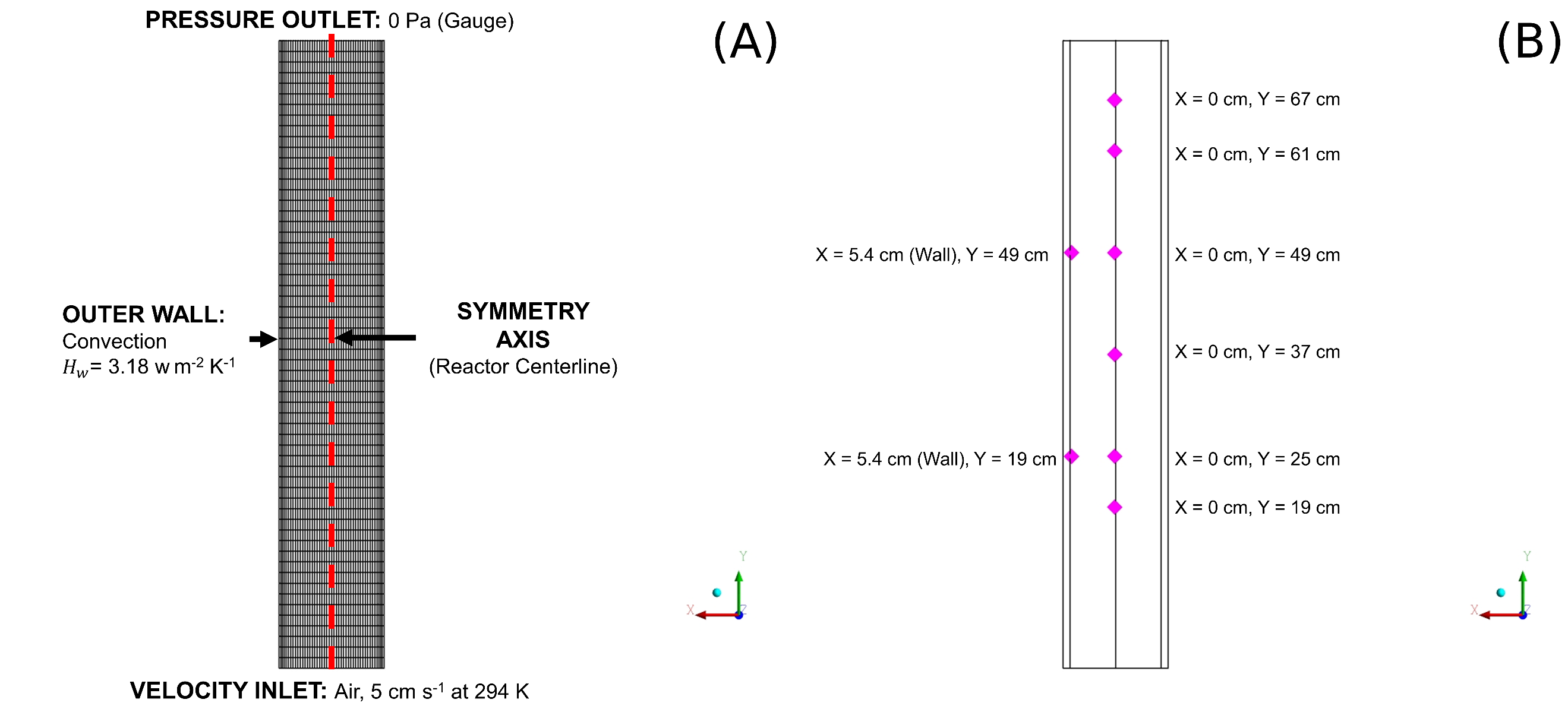


Figure B4: The axisymmetric domain used for the model validation simulation. (A) The cell arrangement (1121 quadrilateral cells in total) within the domain and the primary boundary conditions. (B) pink diamonds indicate thermocouple positions.

Table B2: Summary of Boundary Conditions for the Model Validation Simulation

| **Boundary**  **Type** | **Position** | **Time Interval** | **Momentum Conditions** | | **Thermal Conditions** | |  |
| --- | --- | --- | --- | --- | --- | --- | --- |
|  |  |  | **Gas Zone** | **Solid Zone** | **Gas Zone** | **Solid Zone** | |
| Velocity  Inlet | $y = 0 m$  $0 m\leq x\leq0.054 m$ | $t_{o}\to t_{f}$ | 0.05 cm s^-1^ | - | 294 K | Zero heat flux | |
| Coupled Wall  *(Sand-Wall Interface)* | $0 m\leq y\leq0.74 m$  $x=0.054 m$ | $t_{o}\to t_{f}$ | - | - | - | Coupled | |
| Inner Wall  *(Air-Wall Interface)* | $0 m\leq y\leq0.74 m$  $x=0.054 m$ | $t_{o}\to t_{f}$ | Stationary wall  X,Y zero shear | - | - | - | |
| Outer Wall | $0 m\leq y\leq0.74 m$  $x=0.062 m$ | $t_{o}\to t_{f}$ | - | - | Zero Heat Flux | Convection  ($H_{w}$) | |
| Symmetry | $0 m\leq y\leq0.74 m$  $x=0 m$ | $t_{o}\to t_{f}$ | - | - | - | - | |
| Pressure Outlet | $y = 0.74 m$  $0 m\leq x\leq0.054 m$ | $t_{o}\to t_{f}$ | 0 Pa  (Gauge) | - | 294 K (Backflow) | Zero heat flux | |


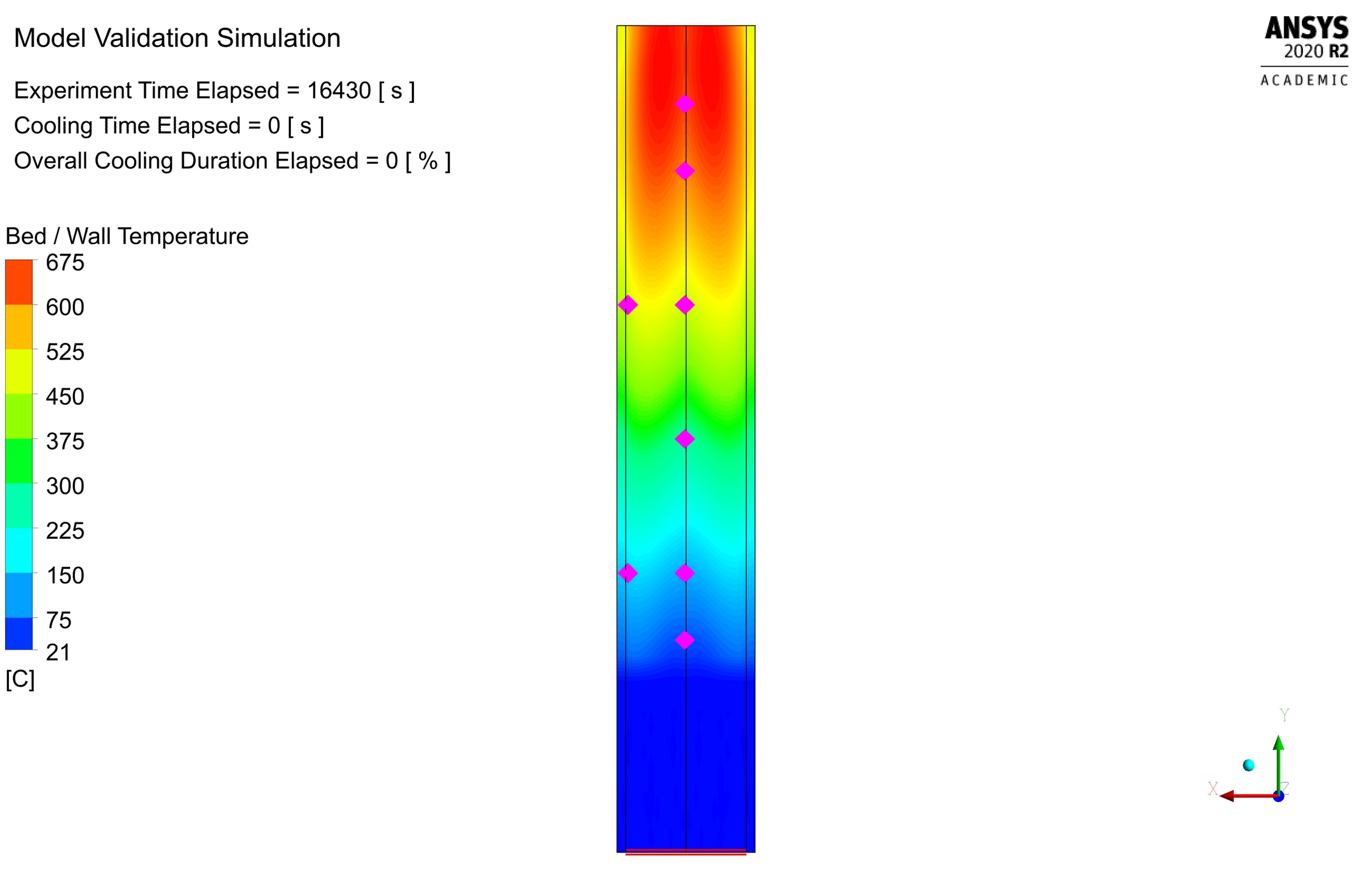


Figure B5: Initial reactor bed temperature distribution for model validation cooling simulation. The red line at the base of domain indicates the smouldering ignition/air injection point.

The initial temperature distribution used the experimental temperature measurements at the start of cooling (i.e., t = 16430s, see Figure B5 above). The experimental spatial coordinates and temperature readings were provided to the MATLAB “Curve Fitting” tool, which returned a characteristic temperature field function which was then input to the model through a user-defined function.

***NOTE: A recording of the Model Validation simulation is seen in the provide video file titled “Model_Validation-Temperature_Distribution”.***

Throughout the simulation, temperature readings were collected from positions within the domain which corresponded to thermocouple positions in the experiment (Figure B4B). The error [%] between the experimental and numerical cooling data was quantified through use of a weighted error function adapted from Zanoni et. al (2019). The modified error function is expressed in Equation B1 which equally weights the Normalized Root-Mean-Squared Deviation (NRMSD) of the wall and centerline temperature data between the experiment and model.

| $Error \left[ \% \right]=\left( 0.5*NRMSD_{cen,avg}+0.5*NRMSD_{wall,avg} \right)\times100$ | (B1) |
| --- | --- |

| $NRMSD_{{cen}/{wall},avg}=\frac{\sum_{j=1}^{n} NRMSD_{{cen}/{wall},j}}{n}$ | (B2) |
| --- | --- |

| $NRMSD_{{cen}/{wall}}=\frac{\sqrt{{\sum_{i=1}^{z} {\left( T_{s,exp}-T_{s,num} \right)_{i}}^{2}}/z}}{{(T}_{s,max}-T_{s,min})}$ | (B3) |
| --- | --- |

In Equation B3 $T_{s,exp}$ and $T_{s,num}$ are the experimental and numerical bed/wall temperatures, respectively. $z$ is the total number of measurements taken over the cooling duration. $T_{s.max}$ and $T_{s,min}$ are the maximum and minimum bed/wall temperatures between both the experimental and numerical data sets. $T_{s,exp}$ and $T_{s,num}$ are compared for each individual measurement, $i$, and at each centerline/wall location. In Equation B2, $n$ is the total number of positions at which temperature readings were taken (wall or centerline) and $j$ indexes the $NRMSD$ calculated for each position.

For the $NRMSD$ centerline calculation ($NRMSD_{cen,avg}$), 6 of the 25 thermocouples were considered (i.e., 19 cm, 25 cm, 37 cm, 49 cm, 61 cm, and 69 cm). Note that for figure clarity, only 4 of these 6 thermocouples (i.e., 25 cm, 37 cm, 49 cm, and 69 cm) are displayed in Figure 2A in the manuscript. The $NRMSD$ wall calculation ($NRMSD_{wall,avg}$) used the wall thermocouples (i.e., $r$ = 5.4 cm) positioned at reactor heights of 25 cm and 49 cm. $NRMSD_{cen,avg}$ and $NRMSD_{wall,avg}$ were calculated as $\sim$6% and $\sim$8%, respectively. This led to an overall simulation error of 7% (Equation B1), as reported in the *Model Validation and Verification* section of the manuscript.

# Supplement C: Model Verification Details

To address solution uncertainty due to spatial discretization, this research followed the Grid Convergence Index (GCI) Method outlined by Celik et al. (2008) and recommended by the *Journal of Fluids Engineering*. For more information regarding this method, the reader is referred to Celik et al. (2008). Summarily, the GCI method calculates a Grid Convergence Index for a given mesh relative to two coarser mesh refinements (i.e., decreased cell count). An acceptable GCI is based on the simulated application as well as user experience. However, a GCI of less than 2% is generally acceptable (Personal Communication, Chris DeGroot) and is used as the threshold in this verification procedure.

As bed cooling time is the focus of this research, the time mass-average temperature of the outlet air to reach 25°C was identified as the critical variable ($\phi_{crit}$), as required for the GCI Method. Two separate procedures were completed for a “fine” (6313 cells) and “coarse” (3393 cells) mesh. For each mesh, two additional meshes of decreased refinement were also considered. The “coarse” mesh was also used for the “fine” mesh GCI procedure, resulting in the use of 5 unique meshes. The Base Case parameters, initial volume-averaged temperature (500°C) and temperature distribution (Cool Edge) were applied for each simulation (see the *Simulation Overviews* section of the manuscript for more details). Following Celik et al. (2008), GCIs of 0.01% and 0.13% were calculated for the “fine” and “coarse” grid, respectively. These GCIs were much less than the 2% threshold and indicate minimal numerical error due to spatial discretization for both grids. This was expected due to the grids being highly uniform and structured, reflected by each grid achieving cell skewness and mesh orthogonality values of essentially 0 and 1, respectively. For computational efficiency, the “coarse” grid was selected as it was composed of approximately a third of the cells of the fine grid and still produced a GCI much less than 2%. The GCI results are summarized below in Table C1.

Table C1: Summary of the Coarse and Fine Grid GCI Procedures

| \| **Coarse Grid***  **GCI Results** \| $\phi_{crit}$ = time required for mass-average outlet air temperature to decline to 25$^{\circ}$C \| \| --- \| --- \| \| *$N_{1}, N_{2}, N_{3}$ \| 3393, 2233, 1273 \| \| $r_{21}$ \| 1.519 \| \| $r_{32}$ \| 1.754 \| \| $\phi_{1}$ \| 7.683 \| \| $\phi_{2}$ \| 7.741 \| \| $\phi_{3}$ \| 7.756 \| \| $p$ \| 4.98 \| \| $\phi_{ext}^{21}$ \| 7.675 \| \| $e_{a}^{21}$ \| 0.75% \| \| $e_{ext}^{21}$ \| 0.11% \| \| $GCI_{fine}^{21}$ \| 0.13% \| | \| **Fine Grid***  **GCI Results** \| $\phi_{crit}$ = time required for mass-average outlet air temperature to decline to 25$^{\circ}$C \| \| --- \| --- \| \| *$N_{1}, N_{2}, N_{3}$ \| 6313, 4753, 3393 \| \| $r_{21}$ \| 1.328 \| \| $r_{32}$ \| 1.401 \| \| $\phi_{1}$ \| 7.611 \| \| $\phi_{2}$ \| 7.605 \| \| $\phi_{3}$ \| 7.683 \| \| $p$ \| 8.13 \| \| $\phi_{ext}^{21}$ \| 7.611 \| \| $e_{a}^{21}$ \| 0.07% \| \| $e_{ext}^{21}$ \| 0.01% \| \| $GCI_{fine}^{21}$ \| 0.01% \| |
| --- | --- | --- | --- | --- | --- | --- | --- | --- | --- | --- | --- | --- | --- | --- | --- | --- | --- | --- | --- | --- | --- | --- | --- | --- | --- | --- | --- | --- | --- | --- | --- | --- | --- | --- | --- | --- | --- | --- | --- | --- | --- | --- | --- | --- | --- | --- | --- | --- | --- |

In the table above, $N$-values are the cell counts of each mesh used in the GCI procedure with $N_{1}$ referring to the highest cell count and $N_{3}$ the lowest. $r$-values are the refinement factors between grids, $\phi$-values are the critical values (mentioned previously) produced from cooling simulations featuring each cell count, $p$ is the apparent order, $\phi_{ext}^{21}$ is the extrapolated solution, $e_{a}^{21}$ is approximate relative error, $e_{ext}^{21}$ is extrapolated relative error, and $GCI_{fine}^{21}$ indicates the fine Grid Convergence Index of the mesh $N_{1}$.

| $h=\frac{1}{N} \sum_{i=1}^{N} \Delta A_{i}$ | (C1) |
| --- | --- |

| $r=\frac{h_{coarse}}{h_{fine}}, r_{32}=\frac{h_{3}}{h_{2}}, r_{21}=\frac{h_{2}}{h_{1}}$ | (C2),(C3),(C4) |
| --- | --- |

| $\varepsilon_{32}= \phi_{3}-\phi_{2}, \varepsilon_{21}= \phi_{2}-\phi_{1}$ | (C5),(C6) |
| --- | --- |

| $p=\frac{1}{ln(r_{21})}\left\vert\ln\left\vert\frac{\varepsilon_{32}}{\varepsilon_{21}} \right\vert+q\left( p \right) \right\vert$ | (C7) |
| --- | --- |

| $q\left( p \right)=\ln\left( \frac{r_{21}^{p}-s}{r_{32}^{p}-s} \right)$ | (C8) |
| --- | --- |

| $s=\left\{ \begin{aligned} 1, &\frac{\varepsilon_{32}}{\varepsilon_{21}}>0 \\ -1, &\frac{\varepsilon_{32}}{\varepsilon_{21}}<0 \end{aligned} \right.$ | (C9) |
| --- | --- |

| $\phi_{ext}^{32}=\frac{r_{32}^{p}\phi_{2}-\phi_{3}}{r_{21}^{p}-1}, \phi_{ext}^{21}=\frac{r_{21}^{p}\phi_{1}-\phi_{2}}{r_{21}^{p}-1}$ | (C10),(C11) |
| --- | --- |

| $e_{a}^{21}=\left\vert\frac{\phi_{1}-\phi_{2}}{\phi_{1}} \right\vert, e_{ext}^{21}=\left\vert\frac{\phi_{ext}^{21}-\phi_{1}}{\phi_{ext}^{21}} \right\vert$ | (C12),(C13) |
| --- | --- |

| $GCI_{fine}^{21}=\left\vert\frac{1.25e_{a}^{21}}{r_{21}^{p}-1} \right\vert$ | (C14) |
| --- | --- |

The equations used to determine each of the variables in Table C1 are presented above in Equations C1 through C14. It is highlighted that $h$ is the average “global” cell size which is used when considering a globally observed variable (i.e., cooling time) as $\phi$-values (Celik et al., 2008).

Grid independence is further demonstrated in Figure C1 which displays negligible cooling time variance relative to mesh cell counts. Overall, results were insensitive to spatial discretization.


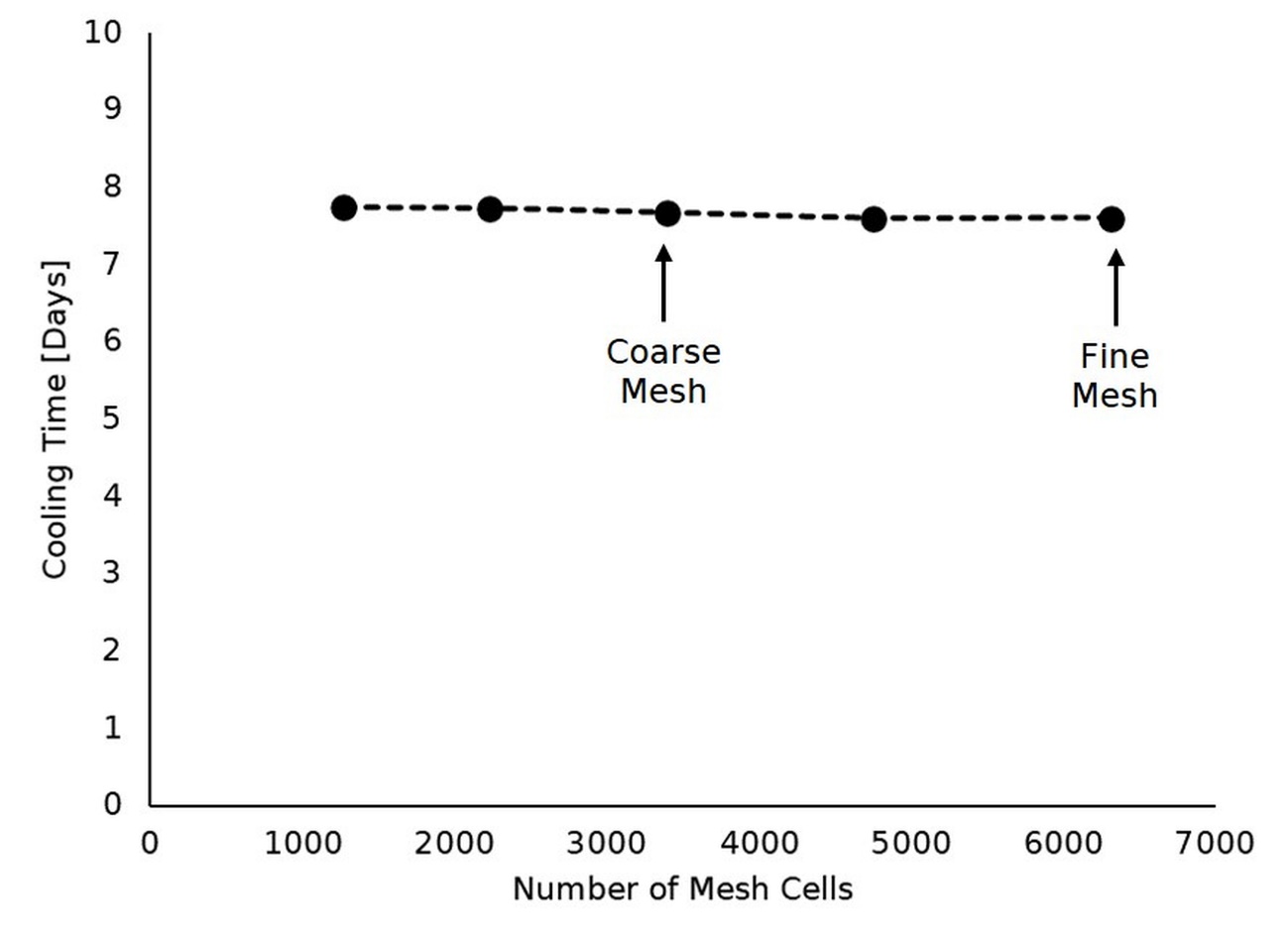


Figure C1: Cooling time versus mesh cell count for each grid used in the GCI procedure. Cooling times for the least to most refined grids (left to right on the plot) were as follows: 7.756 days, 7.741 days, 7.683 days, 7.605, and 7.611 days.

After the optimal mesh cell count was selected, temporal discretization error was then considered. To highlight that results produced from the “coarse” grid were independent of the time stepping method, five additional simulations were completed. For each simulation, the cooling times were again recorded and compared to assess temporal discretization error. The Base Case setup was again used for all simulations. However, of the five completed simulations, only one simulation used the Base Case time stepping method. The remain four simulations *decreased* the Base Case time step size by 75% and 50% as well as *increased* the Base Case time step size by 50% and 100%.

The Base Case time stepping method featured “early” and “late” time step sizes of 50s and 450s, respectively. The purpose of the “early” time step was enhanced solution stability the early stages of the simulation. For all simulations, the “early” time step size was applied for approximately the first 0.5 days of cooling. After which convergence was deemed to be reasonably robust and a larger time step (the “late” time step) was applied in the interest of decreasing computational time. The “late” time step size was then applied for the rest of the simulation duration. Table C2 summarizes the changes of “early” and “late” time step size between each of the completed simulations.

Table C2: “Early” and “Late” Time Step Size for Simulations Investigation Temporal Discretization Error

| **Simulation Designation** | **% Change of Time Step Size from Base Case**  **(Early and Late)** | **“Early”**  **Time Step Size**  **(s)** | **“Late”**  **Time Step Size**  **(s)** |
| --- | --- | --- | --- |
| “Quartered” | -75% | 12.5 | 112.5 |
| “Halved” | -50% | 25 | 225 |
| Base Case | - | 50 | 450 |
| “Plus Half” | +50% | 75 | 577 |
| “Doubled” | +100% | 100 | 900 |

The simulations results are displayed in Figure C2 from which it is evident that cooling times varied negligibly. Therefore, it was concluded that the Base Case time stepping method introduced minimal temporal discretization error to the cooling simulation results. The Base Case time stepping method was used for all further simulations due to its balance of computational efficiency and temporal resolution.


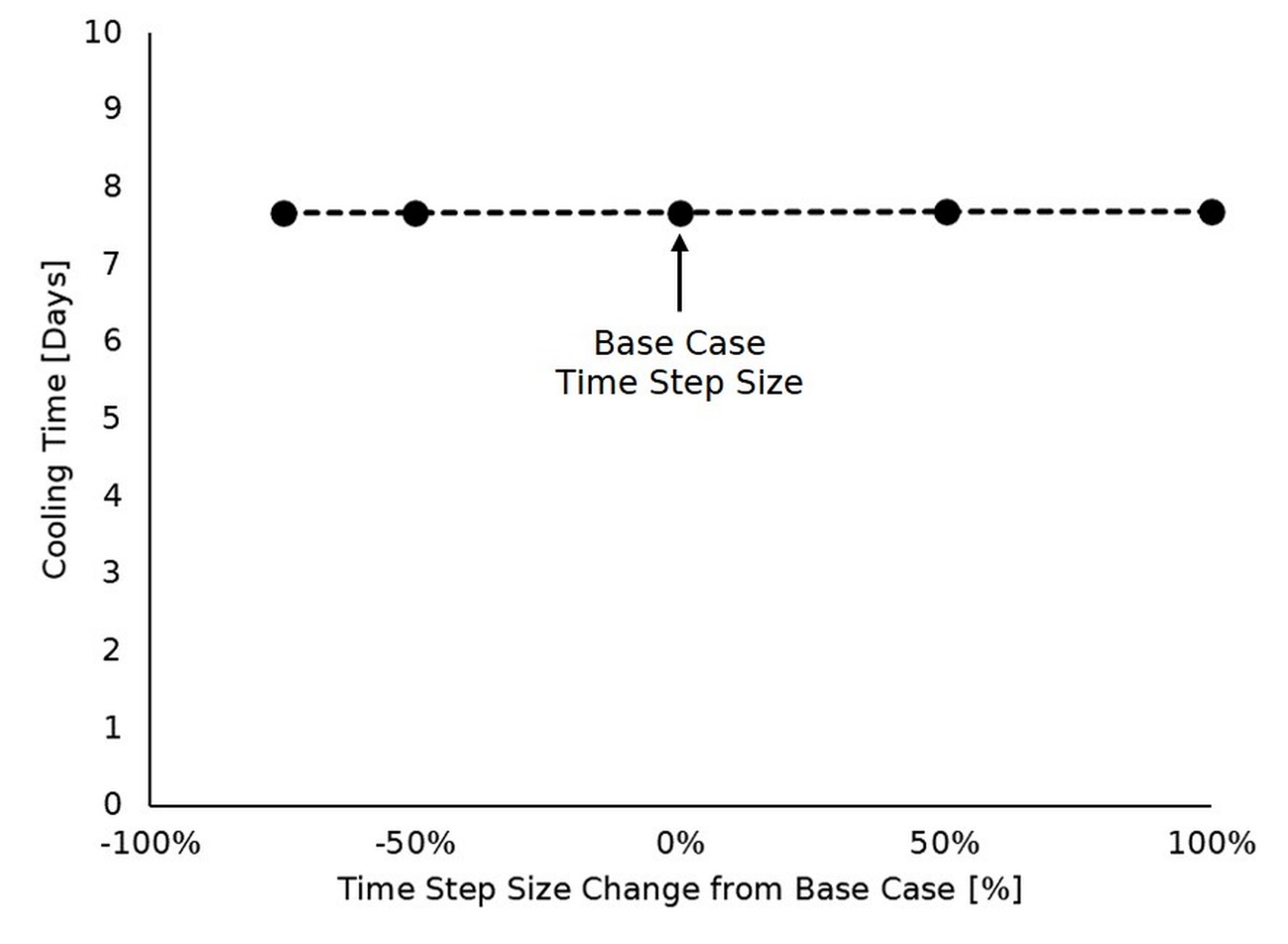


Figure C2: Cooling time versus time step change (%) relative to the Base Case time step sizes. Cooling times produced by the smallest to largest (data points from left to right on the plot) time steps were as follows: 7.680 days, 7.681 days, 7.683 days, 7.689 days, and 7.690 days.

# Supplement D: Compilation of Simulation Results

This section provides compilations of relevant results for all cooling simulations. Results primarily include transient heat losses, energy storage, and sampling location mass flow rate plots. All completed simulations were recorded and are provided as video files. The titles of the video files associated with each simulation are listed within each corresponding section below. Observations and explanations are provided where needed/appropriate. Table D1 below provides a summary of the parameter and cooling time changes between each simulation, relative to the Base Case.

Table D1: Summary of Key Parameter Changes and Results for All Completed Simulations

| **Simulation** | **% Change of Parameters from the Base Case** | | | | **% Change of** $\boldsymbol{t}_{\boldsymbol{c}}$  **from the**  **Base Case** | **Total** $\boldsymbol{t}_{\boldsymbol{c}}$  **[Days]** |
| --- | --- | --- | --- | --- | --- | --- |
|  | $\boldsymbol{T}_{\boldsymbol{s}}^{\boldsymbol{o}}$ | **Initial Temperature**  **Distribution** | $\boldsymbol{\rho}_{\boldsymbol{bs}}$ | $\boldsymbol{q}_{\boldsymbol{g}}$ |  |  |
| **Base Case**  **Parameter Set** | **500 [**$\boldsymbol{^{\circ}}$**C]** | **Cool Edge** | **1660**  **[kg m^-3^]** | **1**  **[cm s^-1^]** | - | **7.7** |
|  | | | | | | |
| Med. Temp. | +50 | - | - | - | +30 | 10.0 |
| High Temp. | +100 | - | - | - | +65 | 12.6 |
| Max. Temp. | +150 | - | - | - | +100 | 15.4 |
|  |  |  |  |  |  |  |
| Homogeneous | - | Homogeneous | - | - | -6 | 7.2 |
| Vertical Gradient | - | Gradient, vertical | - | - | +8 | 8.3 |
| Horizontal Gradient | - | Gradient, horizontal | - | - | +81 | 13.9 |
|  |  |  |  |  |  |  |
| Min. Bulk Density | - | - | -75 | - | -75 | 1.9 |
| Med. Bulk Density | - | - | -50 | - | -50 | 3.9 |
| Max. Bulk Density | - | - | +50 | - | +50 | 11.5 |
|  |  |  |  |  |  |  |
| Med. Air Flux | - | - | - | +50 | -36 | 4.9 |
| High Air Flux | - | - | - | +100 | -53 | 3.6 |
| Max. Air Flux | - | - | - | +200 | -70 | 2.3 |

When considering videos presenting the evolution of the bed pneumatic conductivity ($K_{p}$) distribution it is important to make note of the position and orientation of *higher* $K_{p}$ (lower temperature) layers. Layers of higher pneumatic conductivity which are oriented in the primary direction of the air flow (i.e., from inlet to outlet) are indicative of pathways for thermally-induced preferential air flow. The divergence of air away from lower $K_{p}$, higher temperature zones towards these lower temperature, higher $K_{p}$ pathways lead to longer cooling times, as discussed in the manuscript.

With that said, the influence of air divergence was not analyzed for simulations investigating porous bed bulk density and injection air flux. For both of these simulations sets, all beds were initialized to the Base Case temperature distribution and volume-averaged temperature. Therefore, as air divergence is a *thermally-induced* phenomena, it was expected that these simulations would experience air divergence effects to the same extent as the Base Case. This was confirmed by Figure D14 and Figure D18, in which the distribution of air flow through the bed is similar (if not identical) to the distribution observed during the Base Case simulation (represented by the black line in both figures). As a result, it was concluded that air divergence effects were neither enhanced nor diminished by changes to the bed bulk density or the applied injection air flux.

## Base Case Simulation

**NOTE:** Recordings of the Base Case simulation are seen in the provided video files titled:

1. “Base_Case-Temperature_Distribution”
2. “Base_Case-Pneumatic_Conductivity_Distribution”


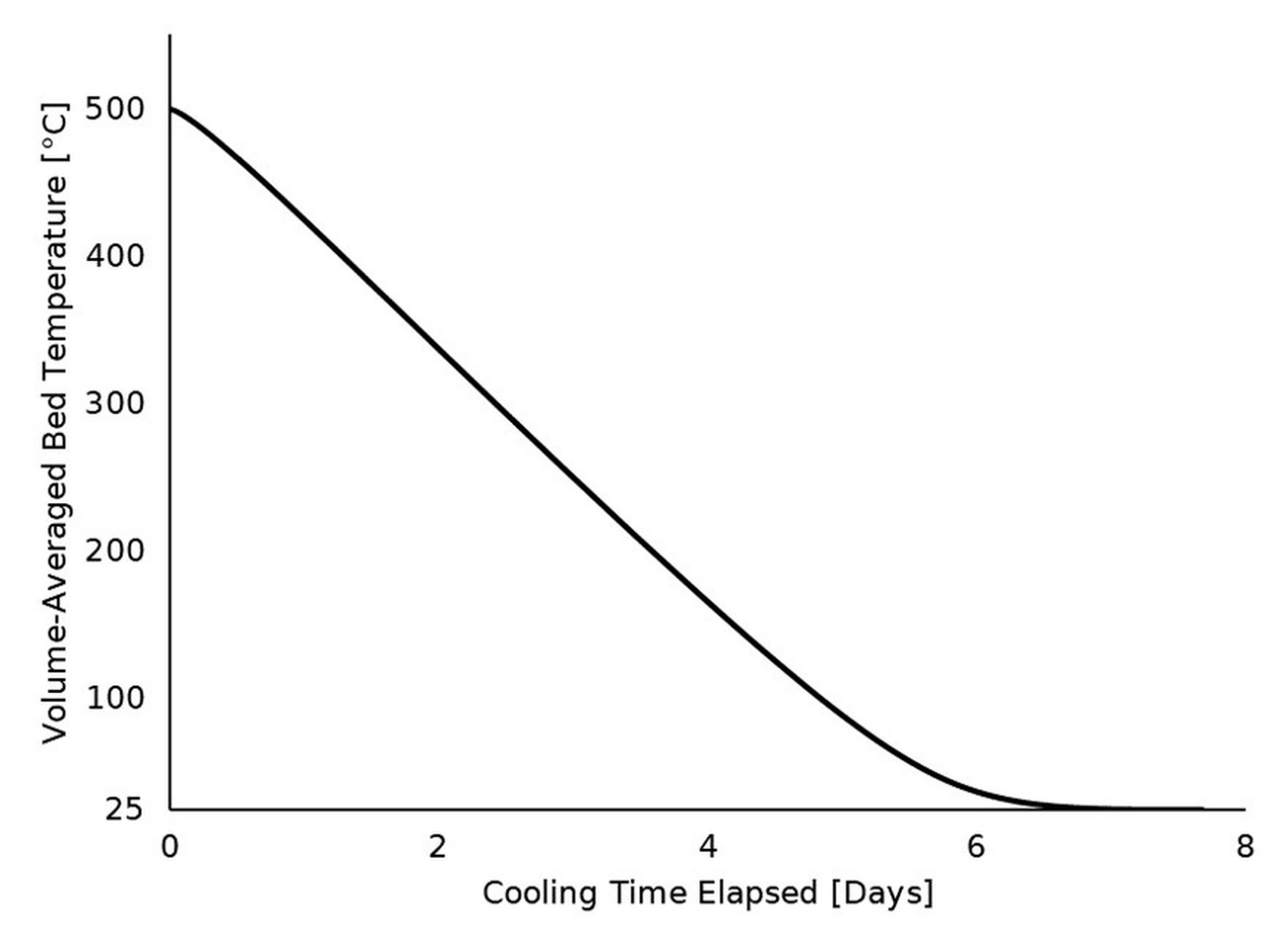


Figure D3: Transient volume-averaged bed temperature of the Base Case simulation.


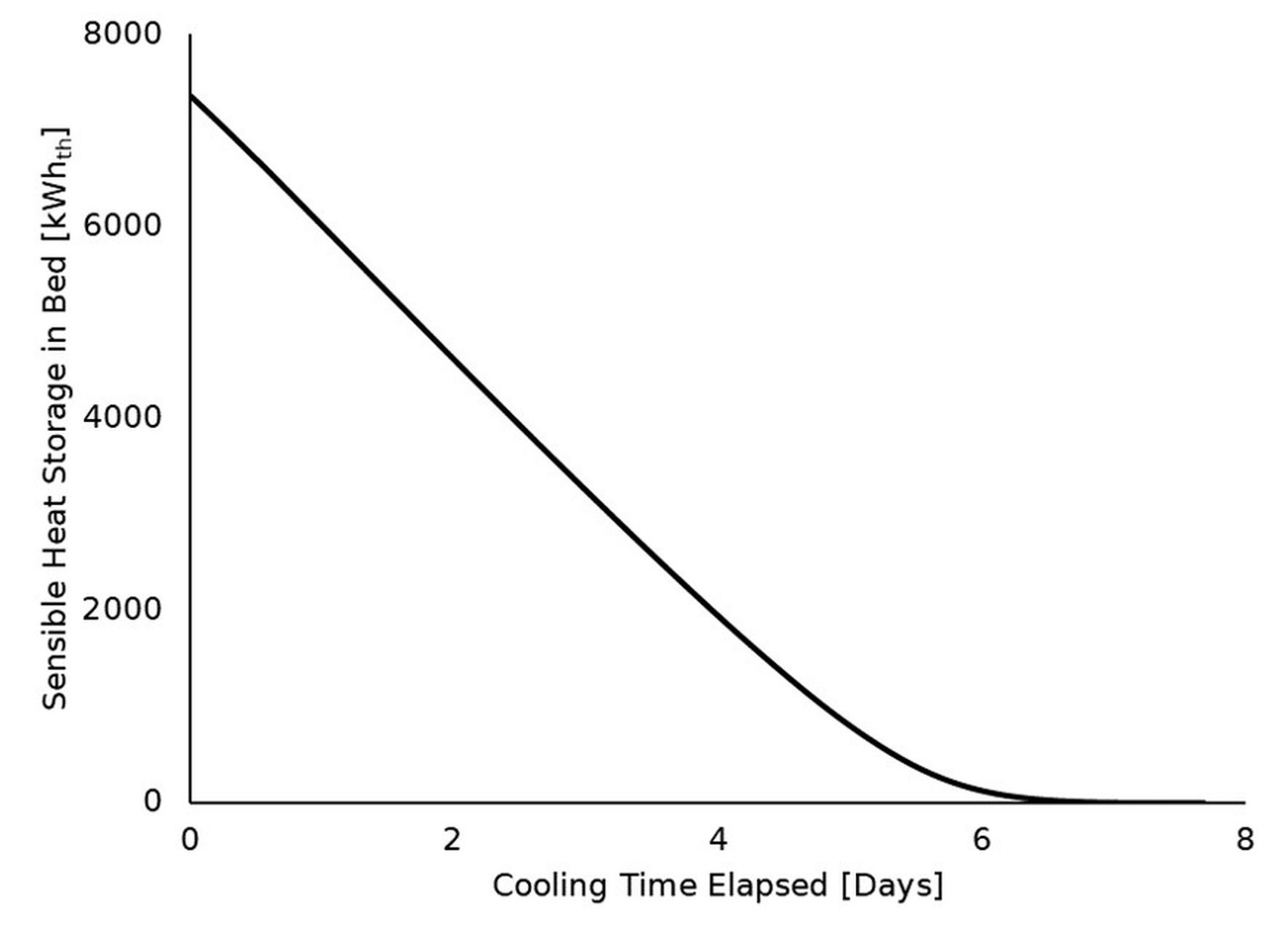


Figure D4: Transient sensible heat storage in the bed for the Base Case simulation


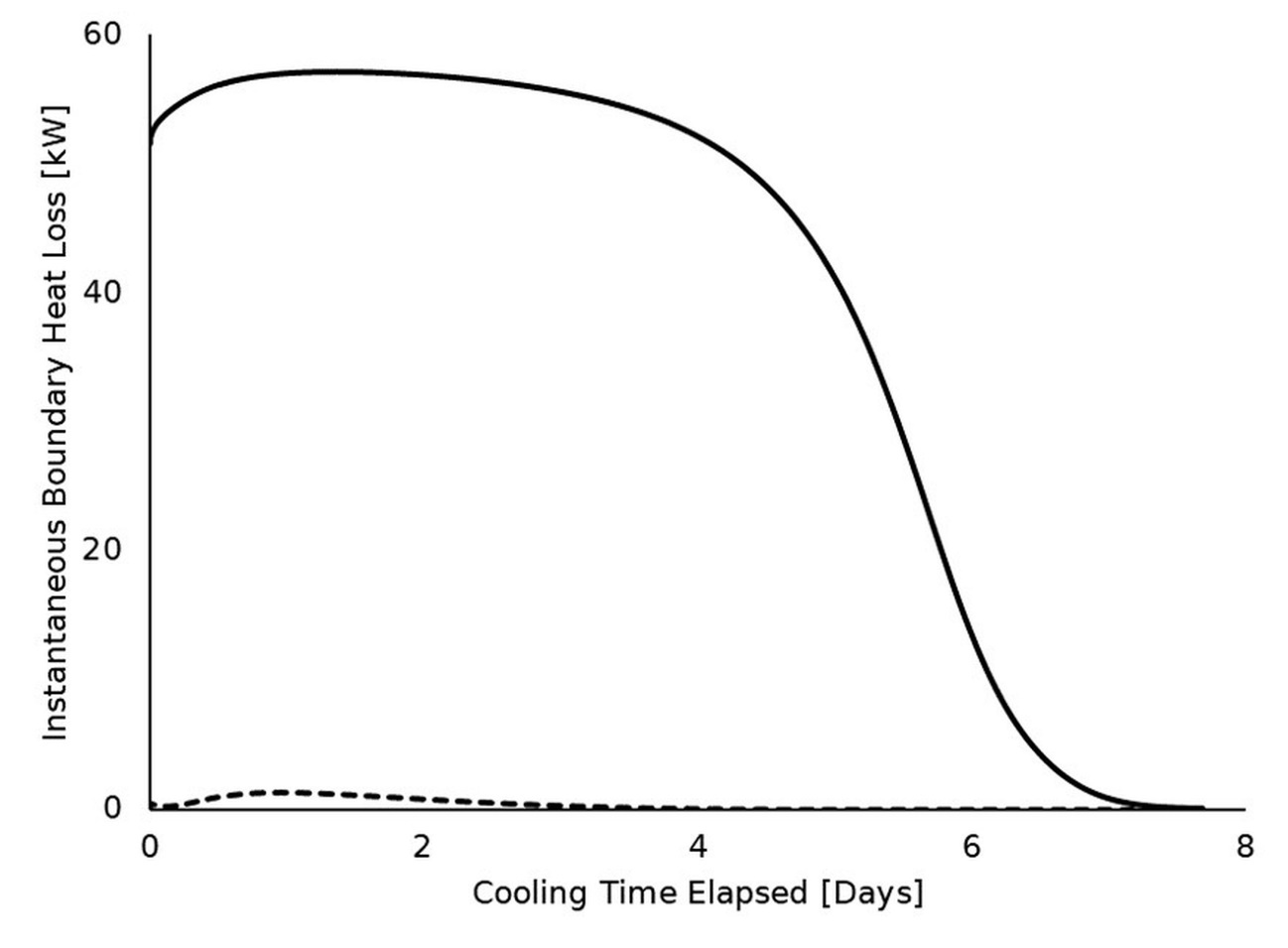


Figure D5: Transient boundary heat losses for the Base Case simulation. The solid and the dotted lines corresponds to outlet and wall heat losses, respectively.

## Initial Volume-Averaged Bed Temperature Simulations

**NOTE:** Recordings of all Initial Volume-Averaged Bed Temperature simulations are seen in the provided video files titled:

1. “Temp_750C-Temperature_Distribution”
2. “Temp_750C-Pneumatic_Conductivity_Distribution”
3. “Temp_1000C-Temperature_Distribution”
4. “Temp_1000C-Pneumatic_Conductivity_Distribution”
5. “Temp_1250C-Temperature_Distribution”
6. “Temp_1250C-Pneumatic_Conductivity_Distribution”

As bed temperature inhomogeneity drives air divergence, it is important to acknowledge the increase of near-wall preferential air flow at higher bed temperatures. Figure D6 plots the resulting total air flow distribution for each initial volume-averaged bed temperature investigated. Greater near-wall air flow was due to the regions closer to the bed center (beneath sampling locations B and C) decreasing in $K_{p}$ as temperature increased.


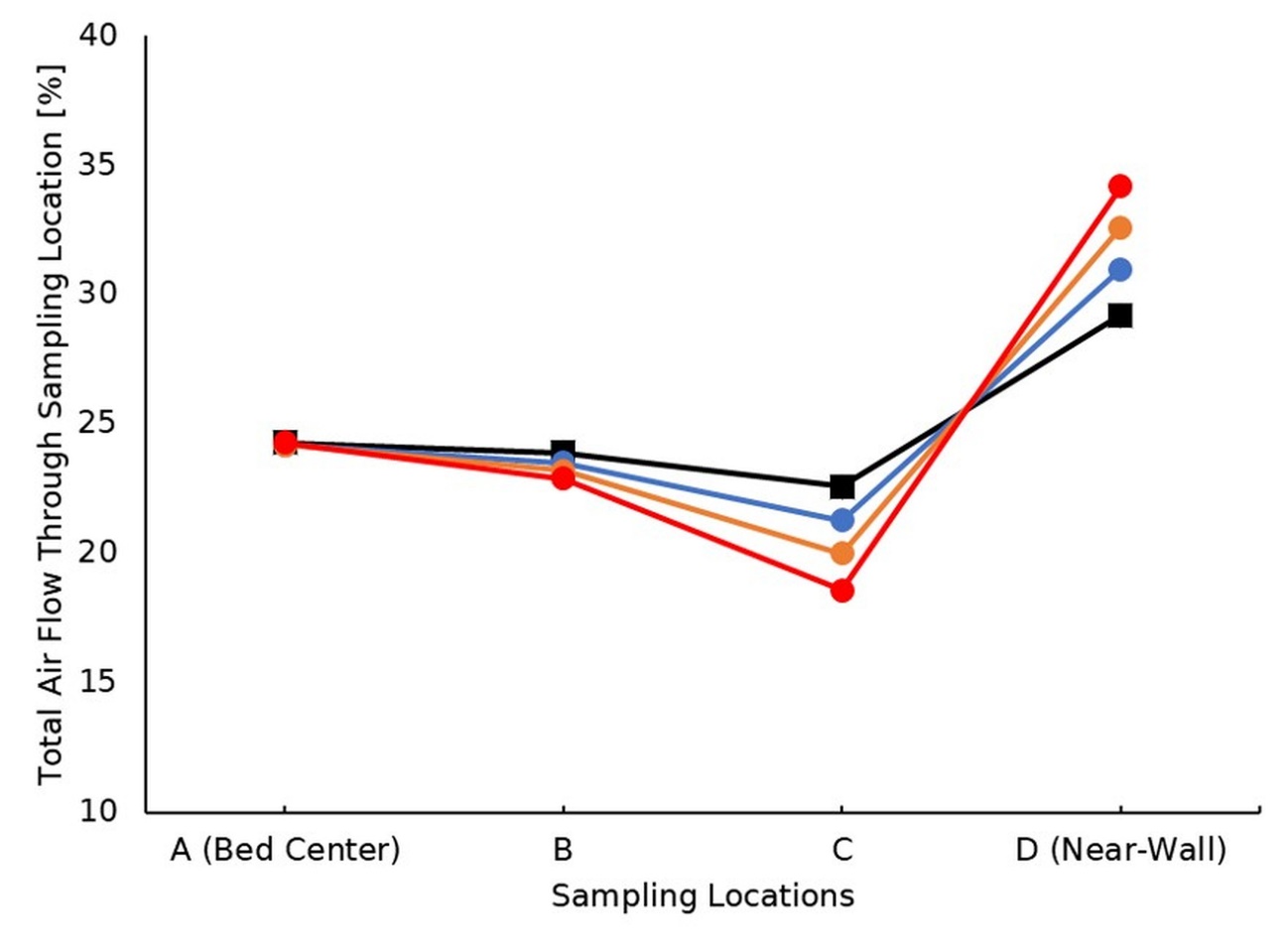


Figure D6: Distribution of total air flow through the outlet sampling locations for each initial volume-averaged bed temperature investigated: 500°C (black line, Base Case), 750°C (blue line, medium temperature), 1000°C (orange line, high temperature), and 1250°C (red line, maximum temperature).

Figure D7 indicates that cooling time was longer for simulations in which greater near-wall air flow was observed (i.e., the higher initial temperature simulations). Figure D7 also present the transient bed temperatures for each initial temperature which resulted from neglecting the air divergence effect (by setting the density and viscosity of air constant at values relative to 25°C). Figure D7 highlights that for each initial temperature, cooling to the ambient temperature was faster when air divergence was not accounted for.


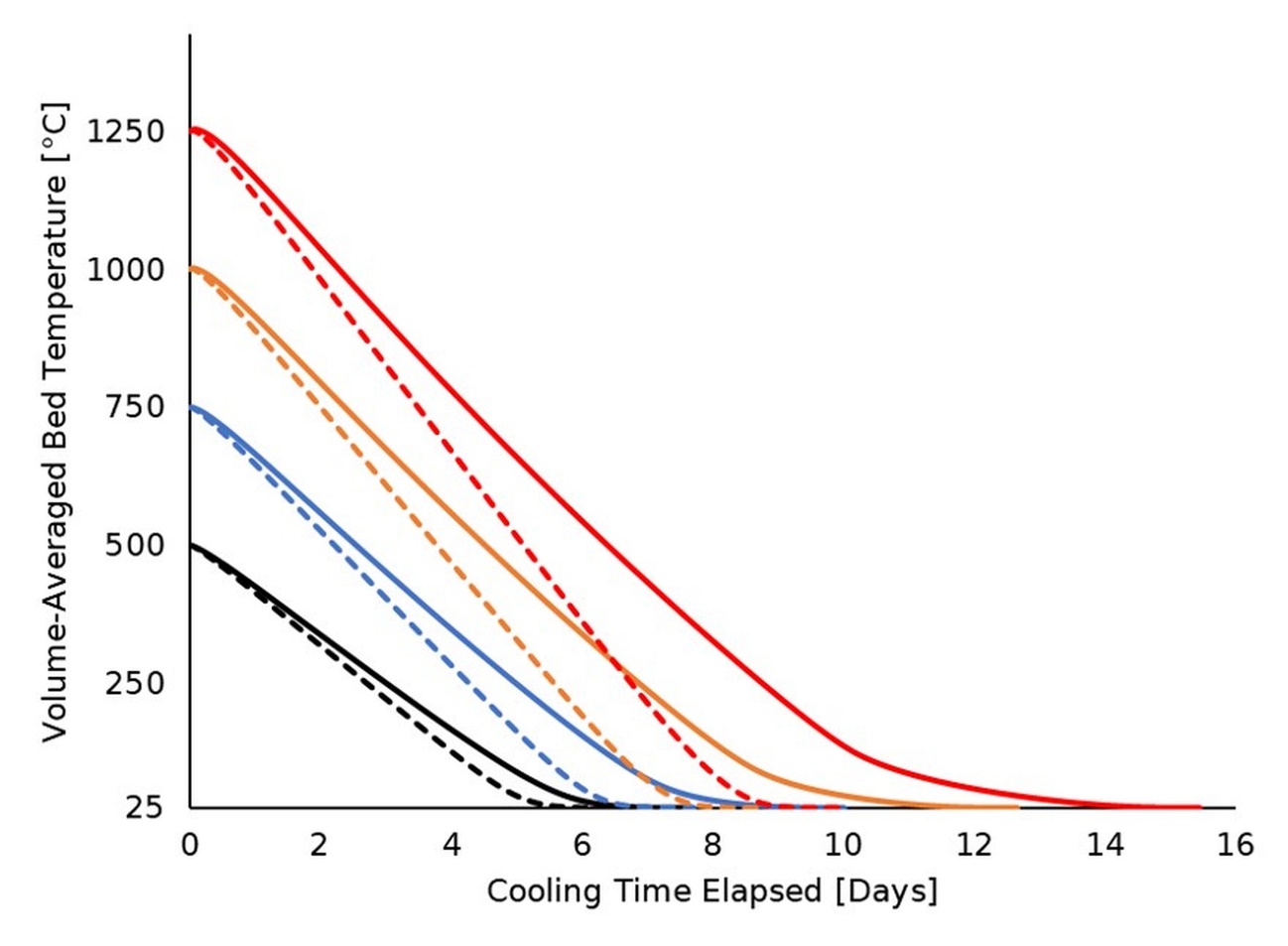


Figure D7: Transient volume-averaged bed temperatures for each initial volume-averaged bed temperature investigated: 500°C (solid black line, Base Case), 750°C (solid blue line, medium temperature), 1000°C (solid orange line, high temperature), and 1250°C (solid red line, maximum temperature). Dashed lines represent simulations in which air divergence effects were neglected.

The *isolated* influence of air divergence on cooling times is directly quantified by the cooling time difference between simulations which accounted for and did not account for air divergence. Therefore, Table D2 below makes evident that at minimum, air divergence accounted for an extra day of cooling (Base Case) and at maximum 5.5 days (maximum initial bed temperature, 1250°C). Both increases are significant extensions of process cycle times. Also presented in Table D2 are the cooling time differences for the simulations investigating the initial bed temperature distribution. These results will be discussed in the next section.

Table D2: Comparison of Simulation Cooling Times with Air Divergence Effects ON or OFF

| **Simulation Sets** | **Individual**  **Simulation Designations** | **Cooling Time** [Days] | | **Difference** [Days] |
| --- | --- | --- | --- | --- |
|  |  | Air Divergence ***ON*** | Air Divergence ***OFF*** |  |
| **Base-Case** | | **7.7** | **6.6** | **1.1** |
|  |  |  |  |  |
| **Initial Volume-Averaged Temperature** | Med. Temp. | 10.0 | 7.7 | 2.3 |
|  | High Temp. | 12.6 | 8.8 | 3.8 |
|  | Max. Temp. | 15.4 | 9.9 | 5.5 |
|  |  |  |  |  |
| **Initial Temperature Distribution** | Homogeneous | 7.2 | 7.2 | 0 |
|  | Vert. Gradient | 8.3 | 7.0 | 1.3 |
|  | Horiz. Gradient | 13.9 | 8.0 | 5.9 |

Figure D7 and Table D2 also provide insight on how the specific heat capacity of the bed material influences cooling times. By neglecting air divergence effects, cooling time was primarily influenced by the increase of sensible heat storage (i.e., the heat load) with greater bed temperatures. Energy storage increased due to the temperature dependence of the Base Case sand specific heat capacity. In Figure D8 cooling times are plotted relative to initial bed temperature and as a result of neglecting air divergence effects. Therefore, the linear increase of cooling time is directly attributable to the specific heat capacity of the Base Case sand. Figure D8 is complementary to Figure D9 in which it is inferable that beds with greater initial energy storage require longer times for their heat load to completely transferred out of the bed during cooling (noting that air divergence effects are accounted for in Figure D9).


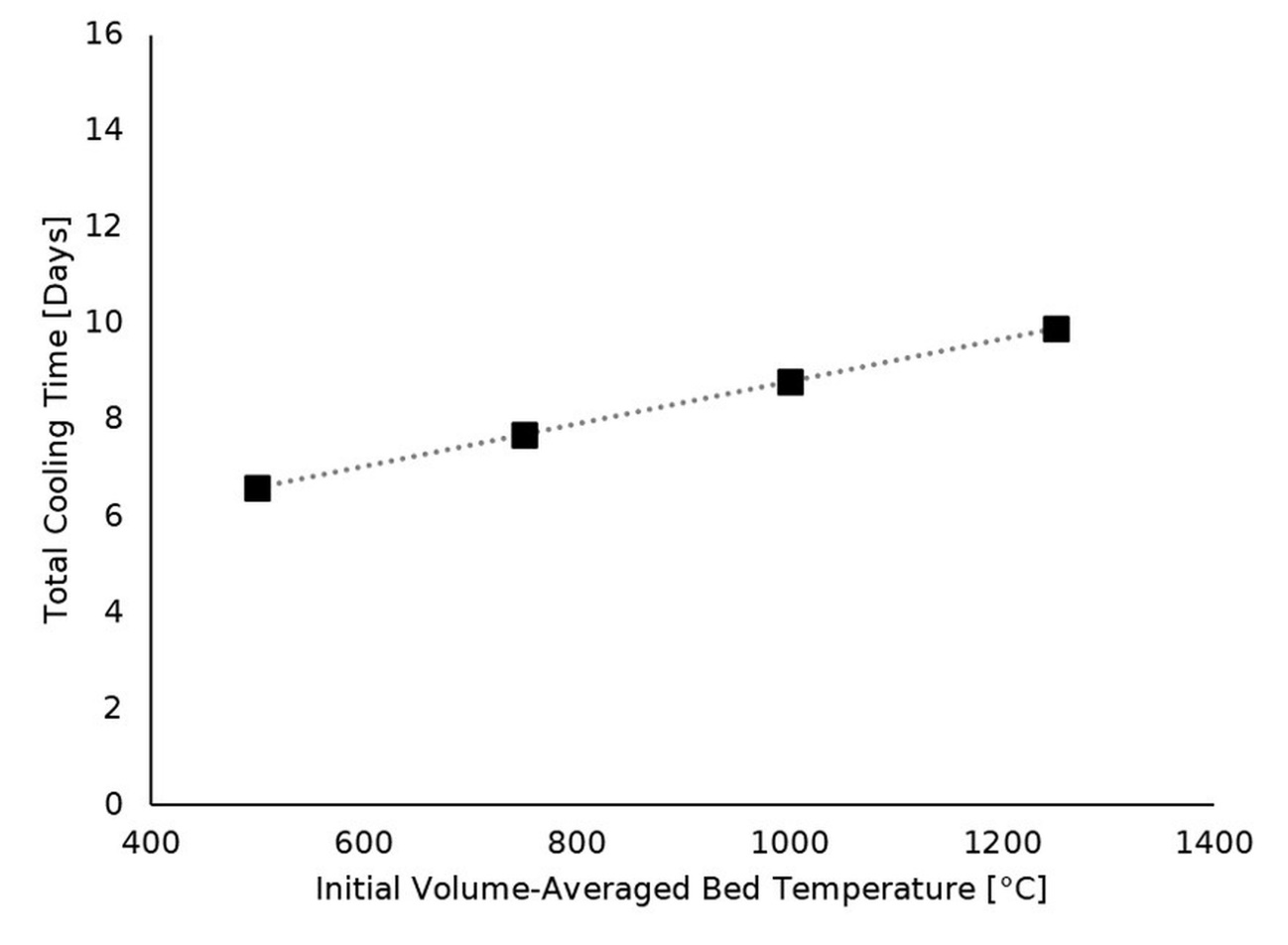


Figure D8: Total cooling times as function of initial volume-averaged bed temperature for simulations in which the effects of air divergence were neglected.


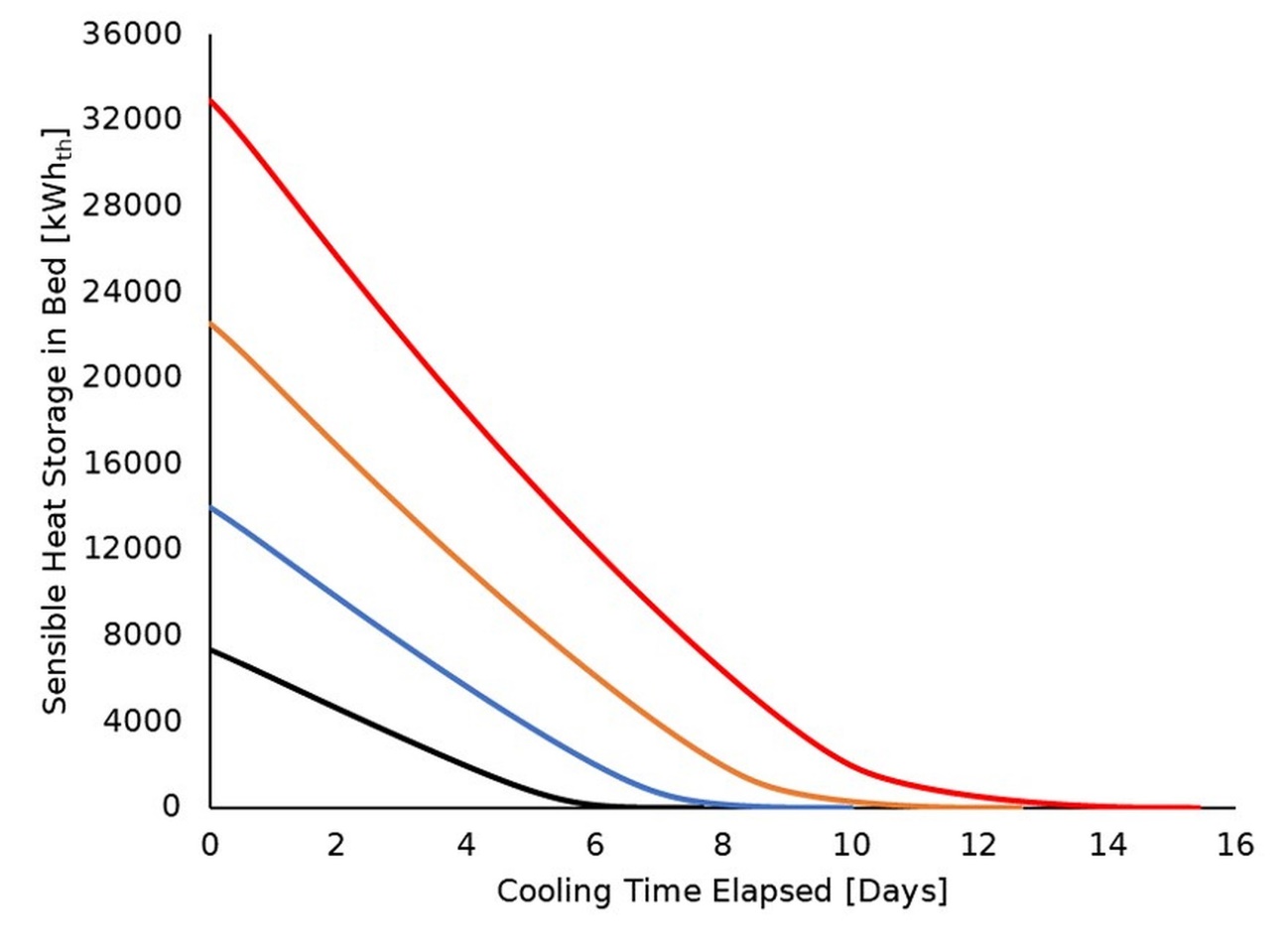


Figure D9: Transient sensible heat storage in the bed for each initial volume-averaged temperature investigated: 500°C (black line, Base Case), 750°C (blue line, medium temperature), 1000°C (orange line, high temperature), and 1250°C (red line, maximum temperature).

This analysis also makes evident that increases of initial bed temperature and the bed bulk density have a similar influence on cooling times. Both parameter variations caused linear increases of cooling time by increasing initial energy storage (i.e., the total heat load).

However, unlike bulk density changes, bed temperature changes are more complex to control. Bed temperatures are determined by smouldering temperatures, which are dependent on a multitude of complex factors (e.g., the energy content of the fuel being smouldered). Therefore, attempting to optimize cooling times through bed temperature control may be extremely impractical, if not impossible. This further highlights the robust cooling time control which bed material selections offers STARx operators.


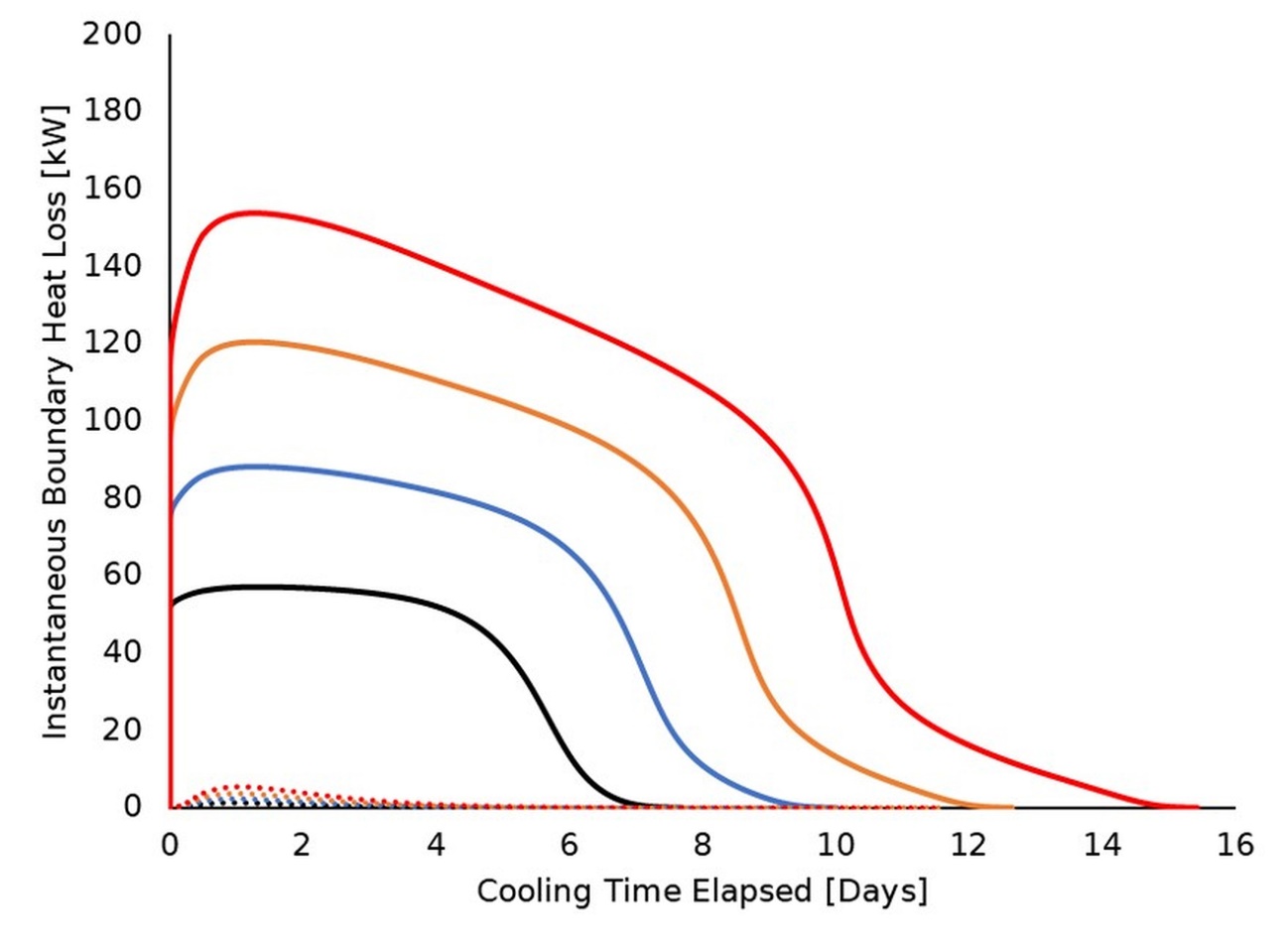


Figure D10: Transient boundary heat losses for each initial volume-averaged temperature investigated: 500°C (black lines, Base Case), 750°C (blue lines, medium temperature), 1000°C (orange lines, high temperature), and 1250°C (red lines, maximum temperature). Solid lines indicate outlet heat losses, dotted lines are wall heat losses.

## Initial Bed Temperature Distribution Simulations

**NOTE:** Recordings of all Initial Bed Temperature Distribution simulations are seen in the provided video files titled:

1. “Dist_Homogeneous-Temperature_Distribution”
2. “Dist_Homogeneous-Pneumatic_Conductivity_Distribution”
3. “Dist_Vertical_Gradient-Temperature_Distribution”
4. Dist_Vertical_Gradient-Pneumatic_Conductivity_Distribution”
5. “Dist_Horizontal_Gradient-Temperature_Distribution”
6. “Dist_Horizontal_Gradient-Pneumatic_Conductivity_Distribution”

| 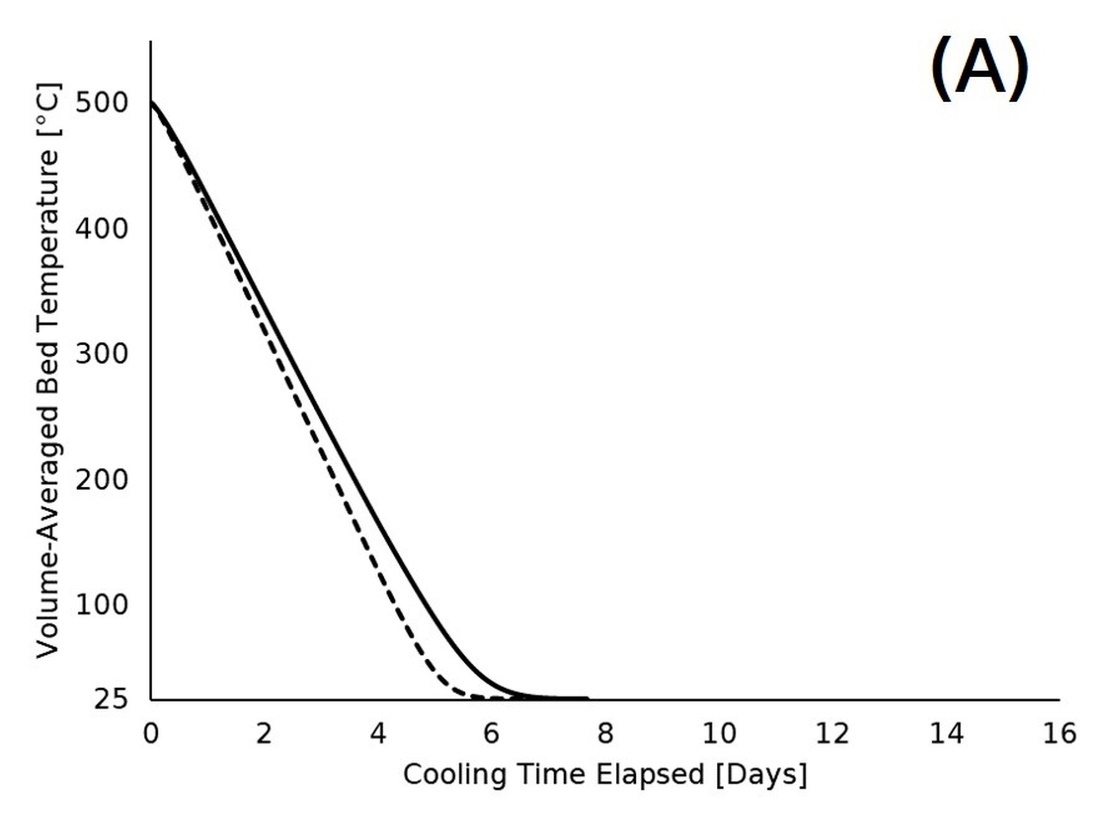 | 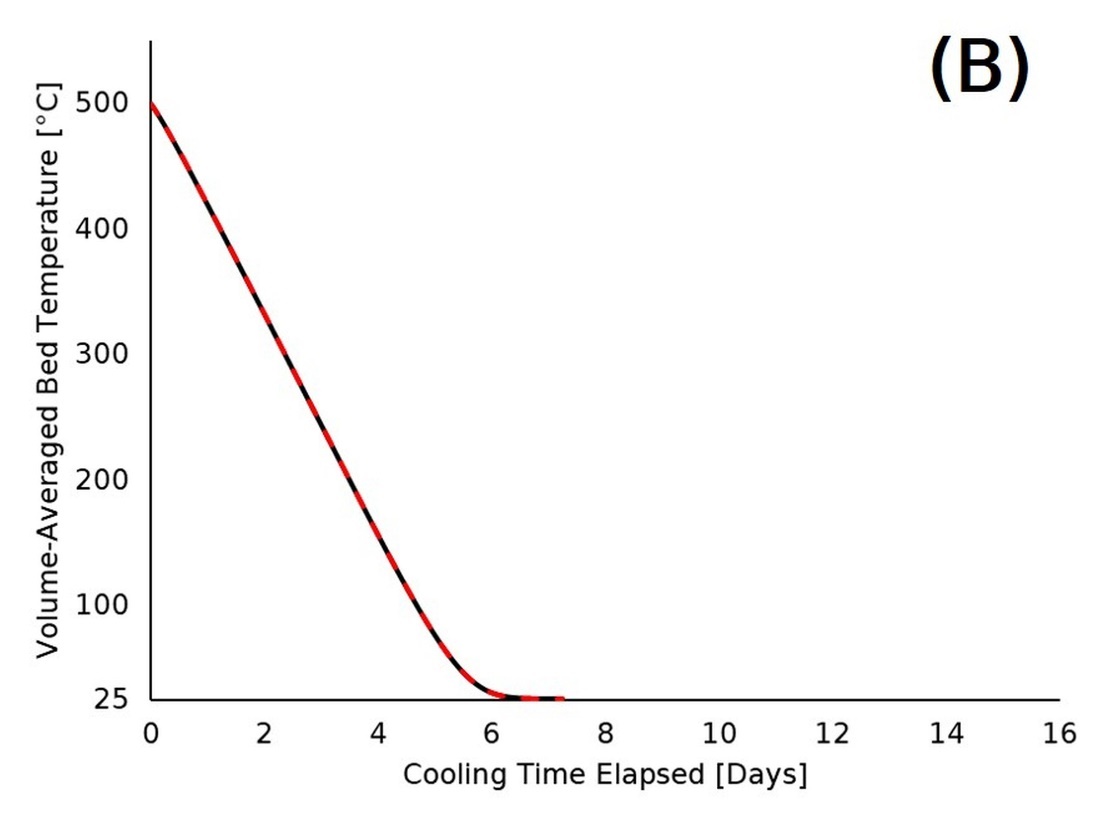 |
| --- | --- |
| 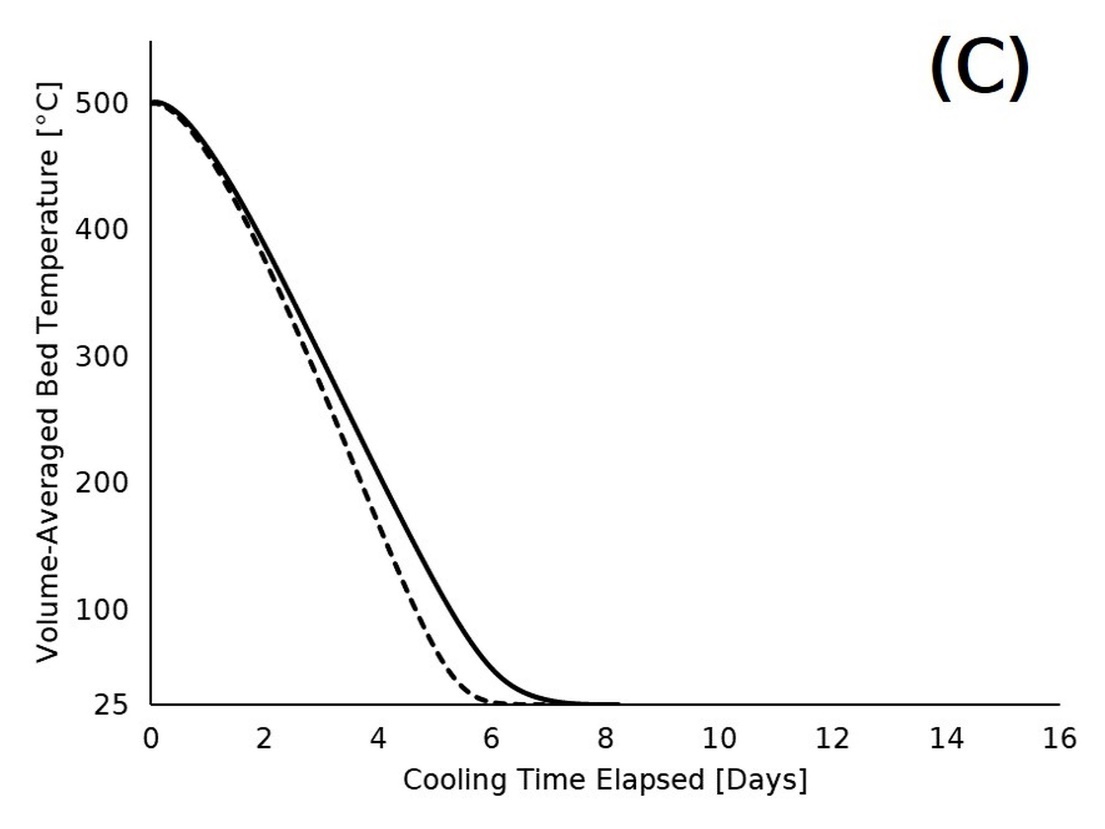 | 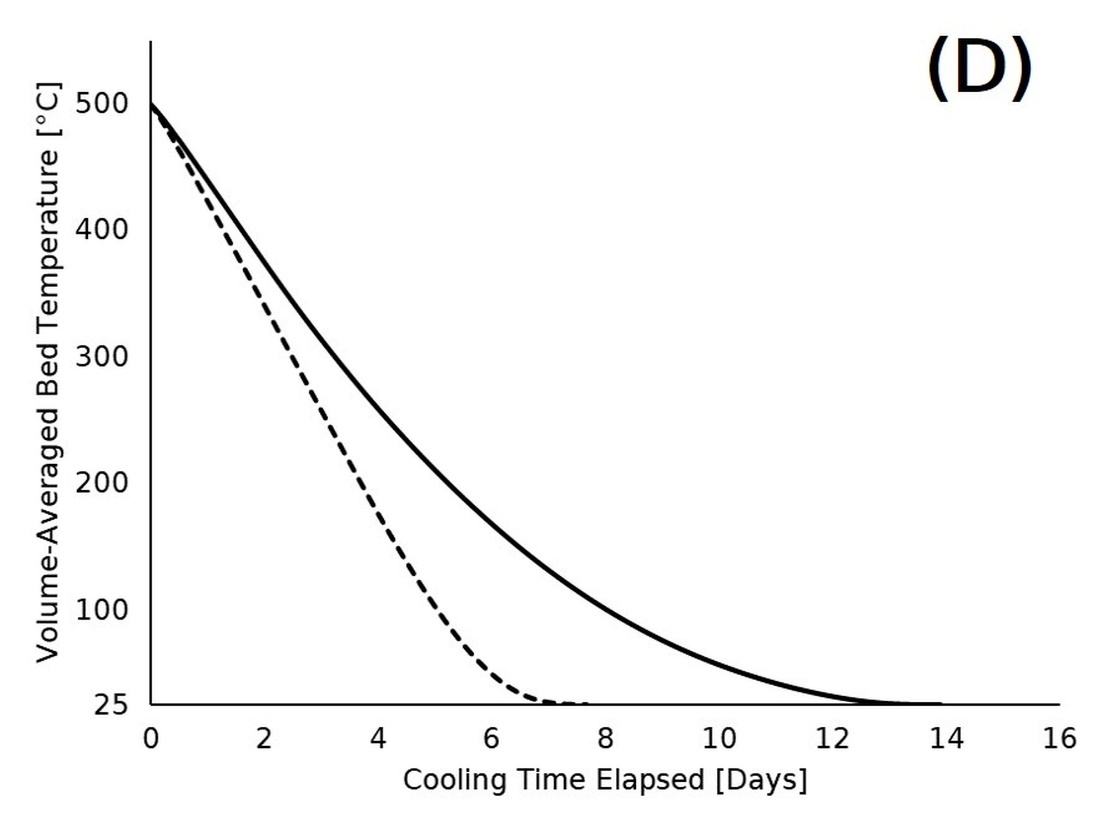 |

Figure D11: Transient volume-averaged bed temperatures for each initial temperature distribution investigated: a) the Cool Edge (Base Case), b) Homogeneous, c) Vertical Gradient, and d) Horizontal Gradient. For each distribution, two sets of bed temperatures are plotted which account for (solid line) and neglect (black dotted line) air divergence effects.

Similar to Figure D7, Figure D11 present transient bed temperatures for the simulations investigating initial bed temperature distribution. Again, for each distribution, simulation results which account for and neglect air divergence effects are compared. The differences in cooling time between each simulation pair are summarized in Table D2. Analysis of Table D2 and Figure D11 further supports how different initial temperature distributions influence the extent of air divergence and the total cooling time (as discussed in *Initial Temperature Distribution* section of the manuscript).

Figure D11 shows that when air divergence is neglected, each initial temperature distribution resulted in a cooling time of approximately 6-7 days. However, when air divergence is considered, the Horizontal Gradient led to a significantly longer cooling time as it featured extensive *lateral* temperature variation. Table D2 indicates that a cooling time difference of 5.9 days was observed between the versions of the Horizontal Gradient simulation which accounted for and neglected air divergence effects. This cooling time difference is contextualized by considering that for the maximum bed temperature (1250°C), air divergence accounted for 5.4 days of the total cooling duration (Table D2). Therefore, air divergence due to the Horizontal Gradient temperature distribution was effectively equivalent to increasing the bed temperature by 150%, although the initial volume-averaged bed temperature was only 500°C.

Conversely, Figure D11 and Table D2 indicate that the Homogeneous simulation resulted in the same cooling time regardless of if air divergences effects were accounted for. Therefore, it can be concluded that complete bed temperature homogeneity (at the start of cooling) will result in the shortest cooling duration relative to a given volume-averaged bed temperature. This further supports the need for STARx optimizations which increase temperature homogeneity in the bed during the smouldering phase, ahead of the start of cooling (as discussed in the *Optimizations for Cooling Time Reduction* section of the manuscript).


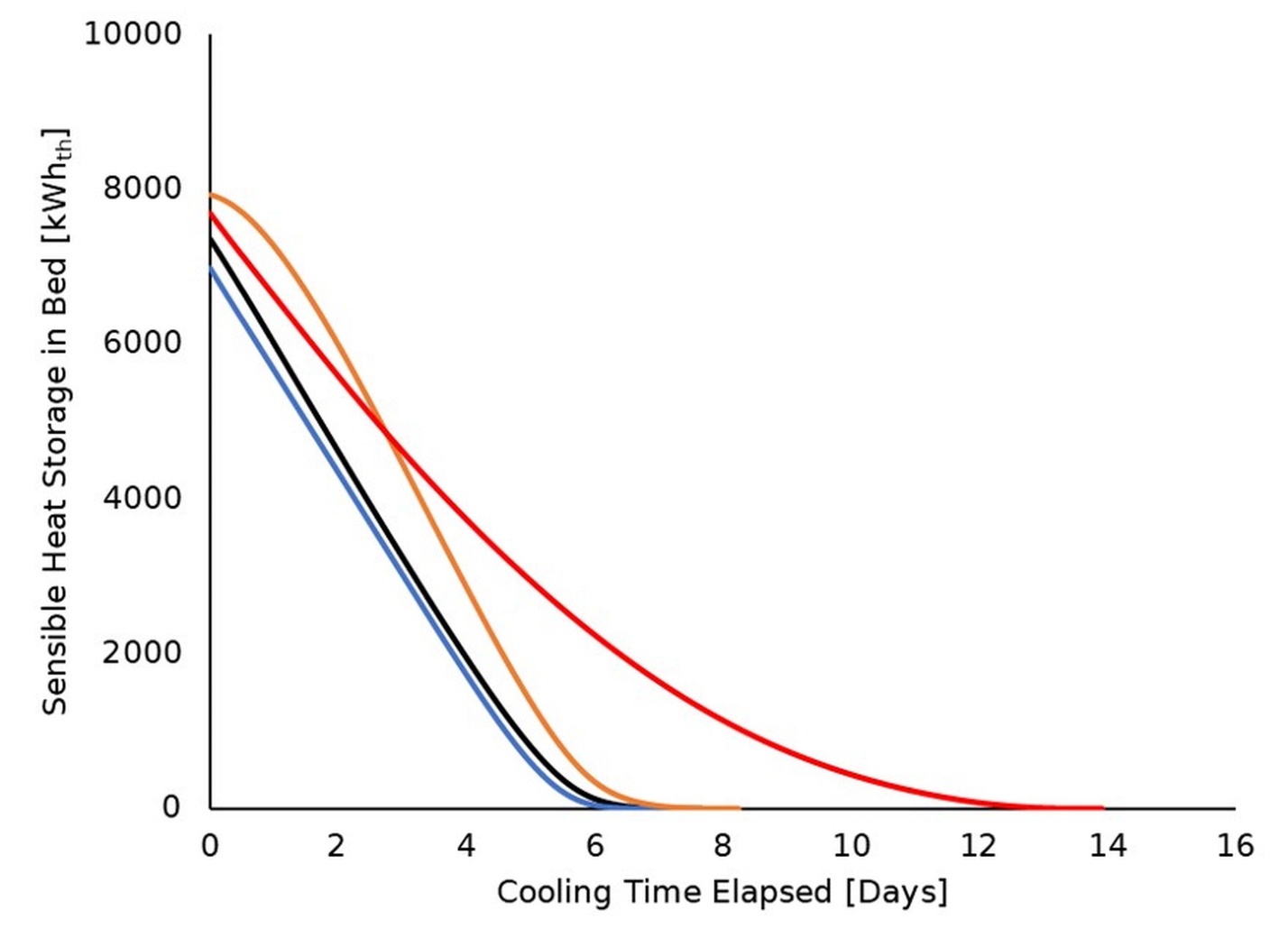


Figure D12: Transient sensible heat storage in the bed for each initial temperature distribution investigated: Cool Edge (black line, Base Case), Homogenous (blue line), Vertical Gradient (orange line), and Horizontal Gradient (red line).


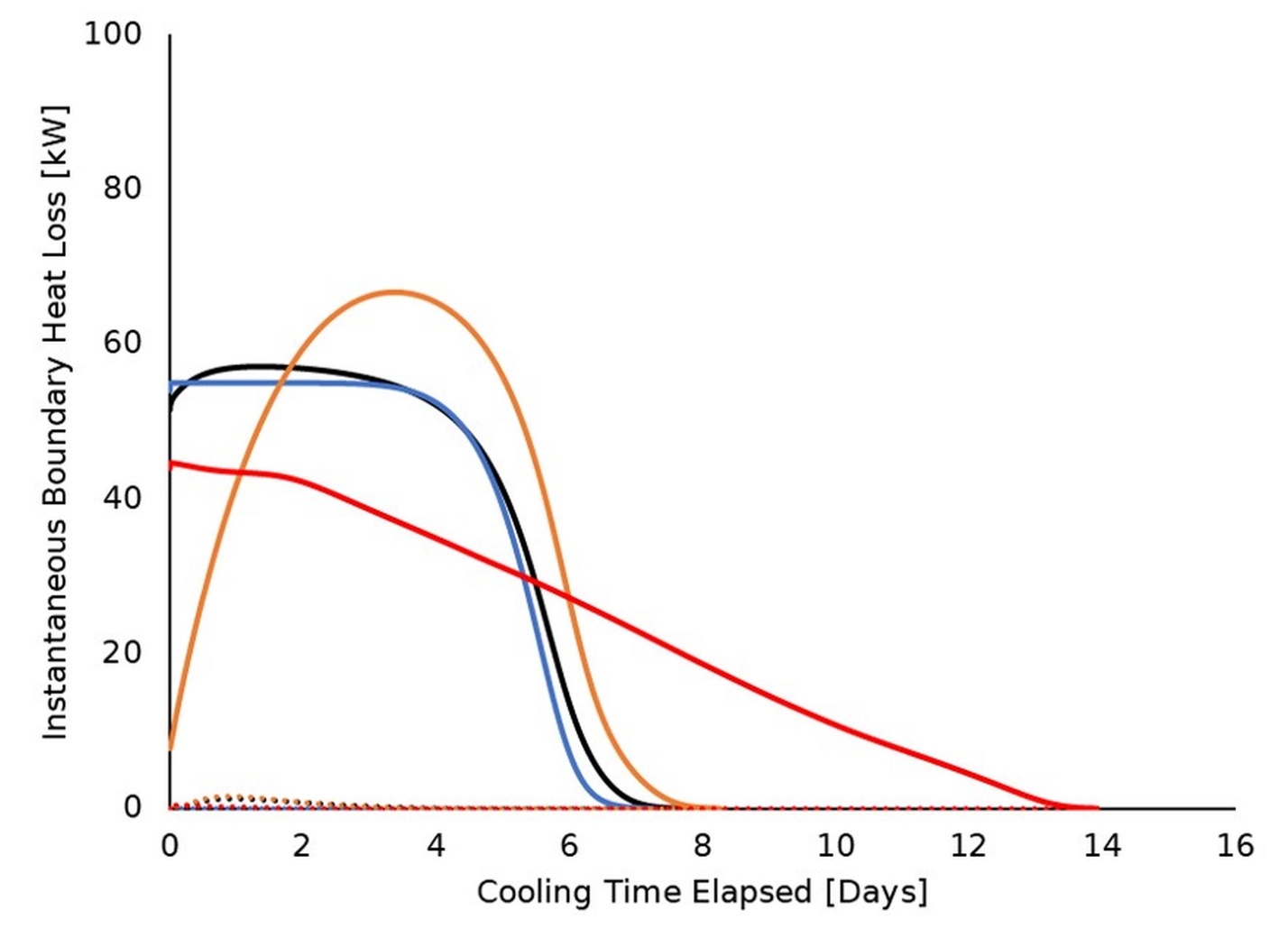


Figure D13: Transient boundary heat losses for each initial temperature distribution investigated: Cool Edge (black line, Base Case), Homogenous (blue line), Vertical Gradient (orange line), and Horizontal Gradient (red line). Solid lines indicate outlet heat losses, dotted lines are wall heat losses.

## Porous Bed Bulk Density Simulations

**NOTE:** Recordings of all Porous Bed Bulk Density simulations are seen in the provided video files titled:

1. “Bulk_Density_415-Temperature_Distribution”
2. “Bulk_Density_415-Pneumatic_Conductivity_Distribution”
3. “Bulk_Density_830-Temperature_Distribution”
4. “Bulk_Density_830-Pneumatic_Conductivity_Distribution”
5. “Bulk_Density_2490-Temperature_Distribution”
6. “Bulk_Density_2490-Pneumatic_Conductivity_Distribution”


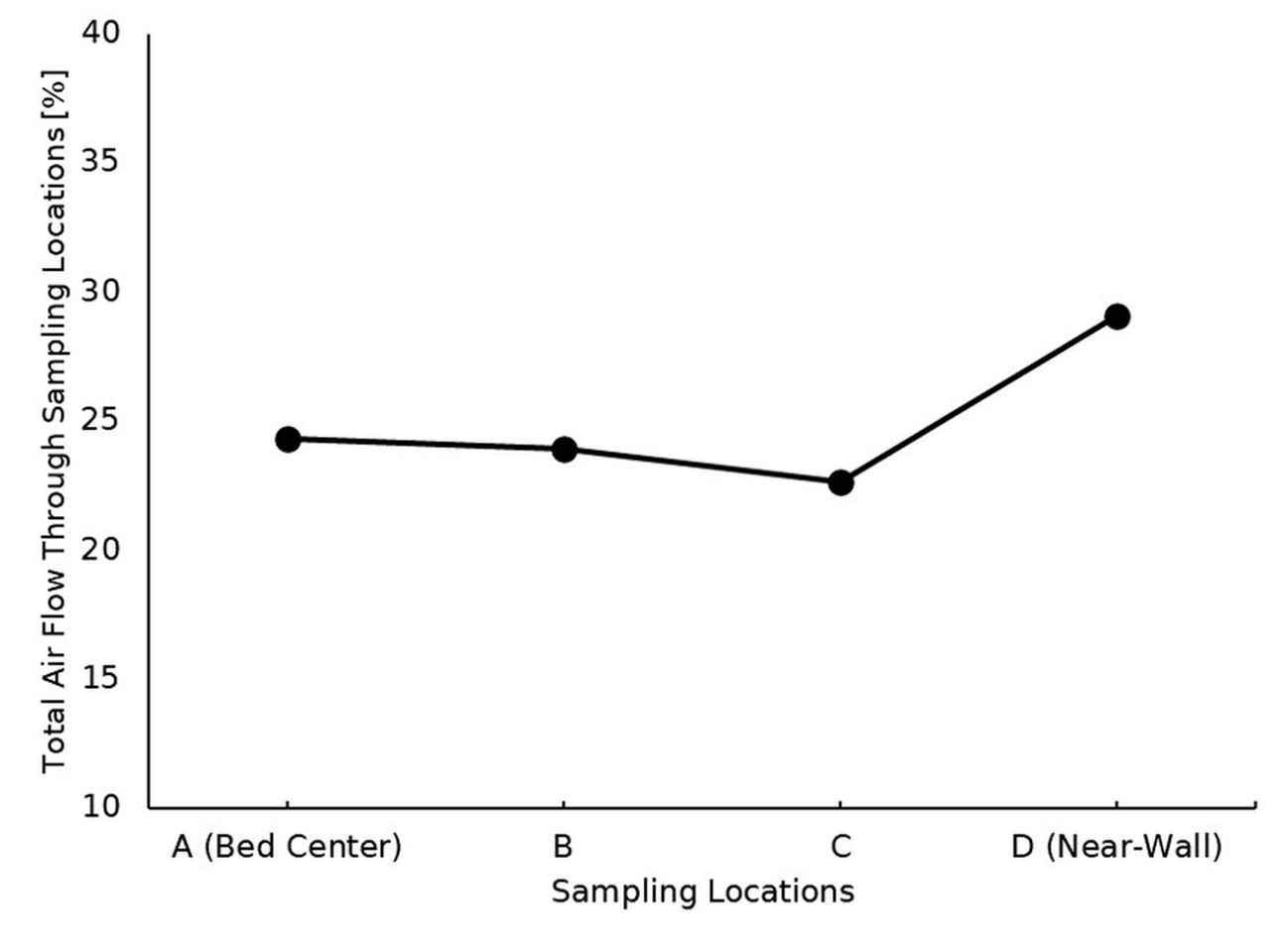


Figure D14: Distribution of total air flow through the outlet sampling locations for all bed bulk density investigated: 415 kg m^-3^ (minimum bulk density), 830 kg m^-3^ (medium bulk density), 1660 kg m^-3^ (Base Case), and 2490 kg m^-3^ (maximum bulk density).


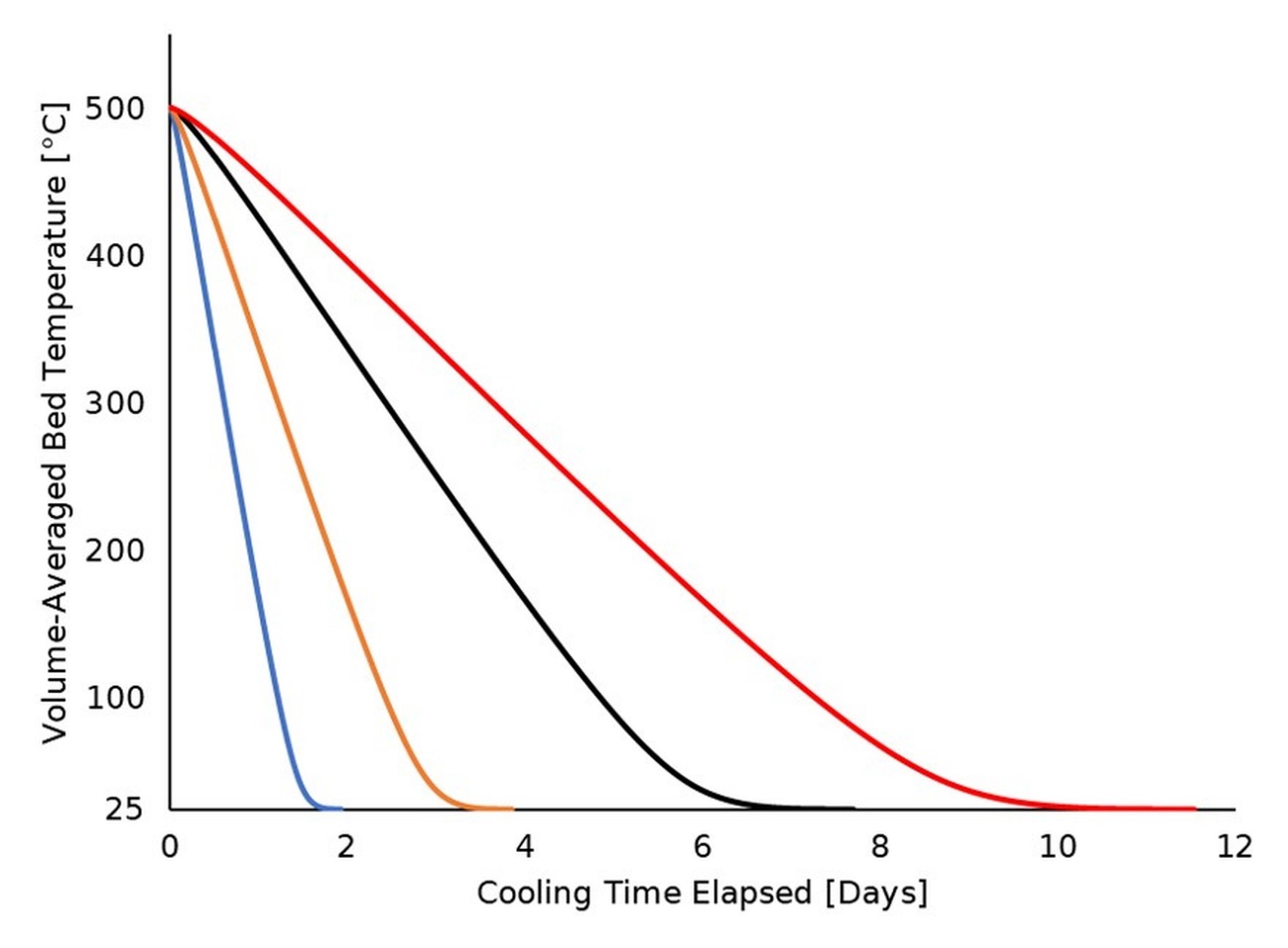


Figure D15: Transient volume-averaged bed temperatures for each porous bed bulk density investigated: 415 kg m^-3^ (blue line, minimum bulk density), 830 kg m^-3^ (orange line, medium bulk density), 1660 kg m^-3^ (black line, Base Case bulk density), and 2490 kg m^-3^ (red line, maximum bulk density).


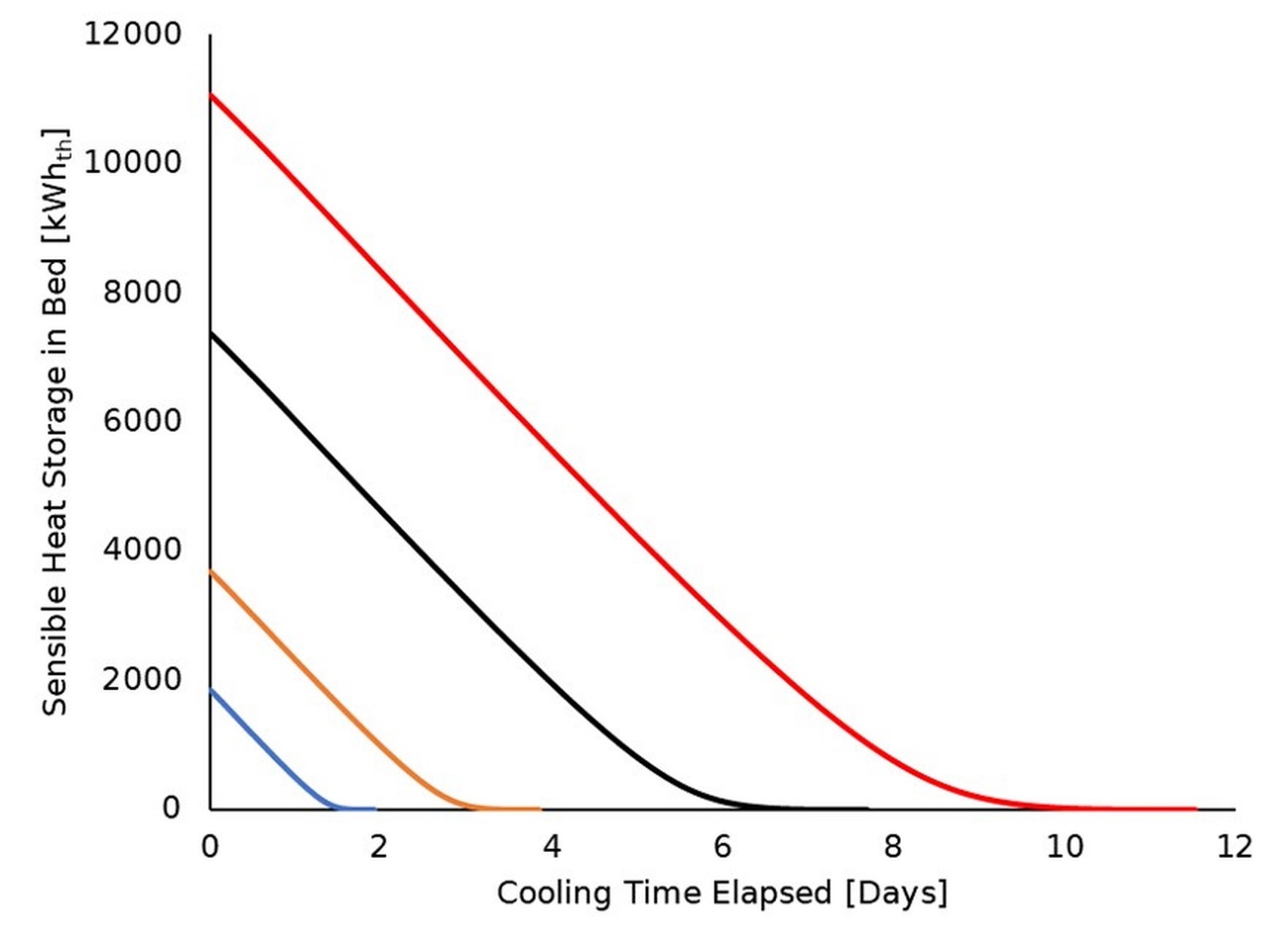


Figure D16: Transient sensible heat storage in the bed for each porous bed bulk density investigated: 415 kg m^-3^ (blue line, minimum bulk density), 830 kg m^-3^ (orange line, medium bulk density), 1660 kg m^-3^ (black line, Base Case bulk density), and 2490 kg m^-3^ (red line, maximum bulk density).


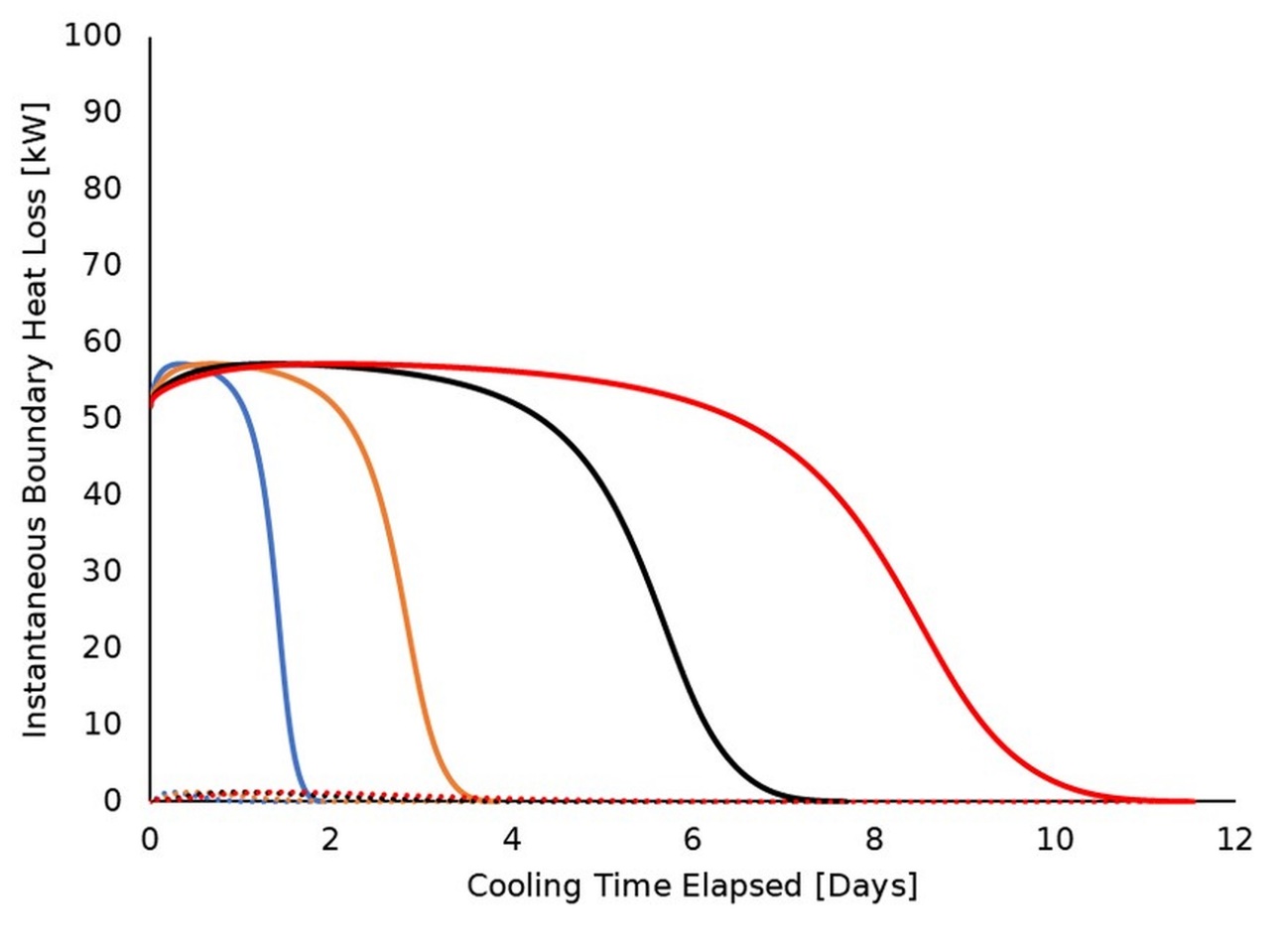


Figure D17: Transient boundary heat losses for each porous bed bulk density investigated: 415 kg m^-3^ (blue line, minimum bulk density), 830 kg m^-3^ (orange line, medium bulk density), 1660 kg m^-3^ (black line, Base Case bulk density), and 2490 kg m^-3^ (red line, maximum bulk density). Solid lines indicate outlet heat losses, dotted lines are wall heat losses.

## Injection Air Flux Simulations

**NOTE:** Recordings of all Injection Air Flux simulations are seen in the provided video files titled:

1. “Air_Flux_1.5-Temperature_Distribution”
2. “Air_Flux_1.5-Pneumatic_Conductivity_Distribution”
3. “Air_Flux_2.0-Temperature_Distribution”
4. “Air_Flux_2.0-Pneumatic_Conductivity_Distribution”
5. “Air_Flux_3.0-Temperature_Distribution”
6. “Air_Flux_3.0-Pneumatic_Conductivity_Distribution”


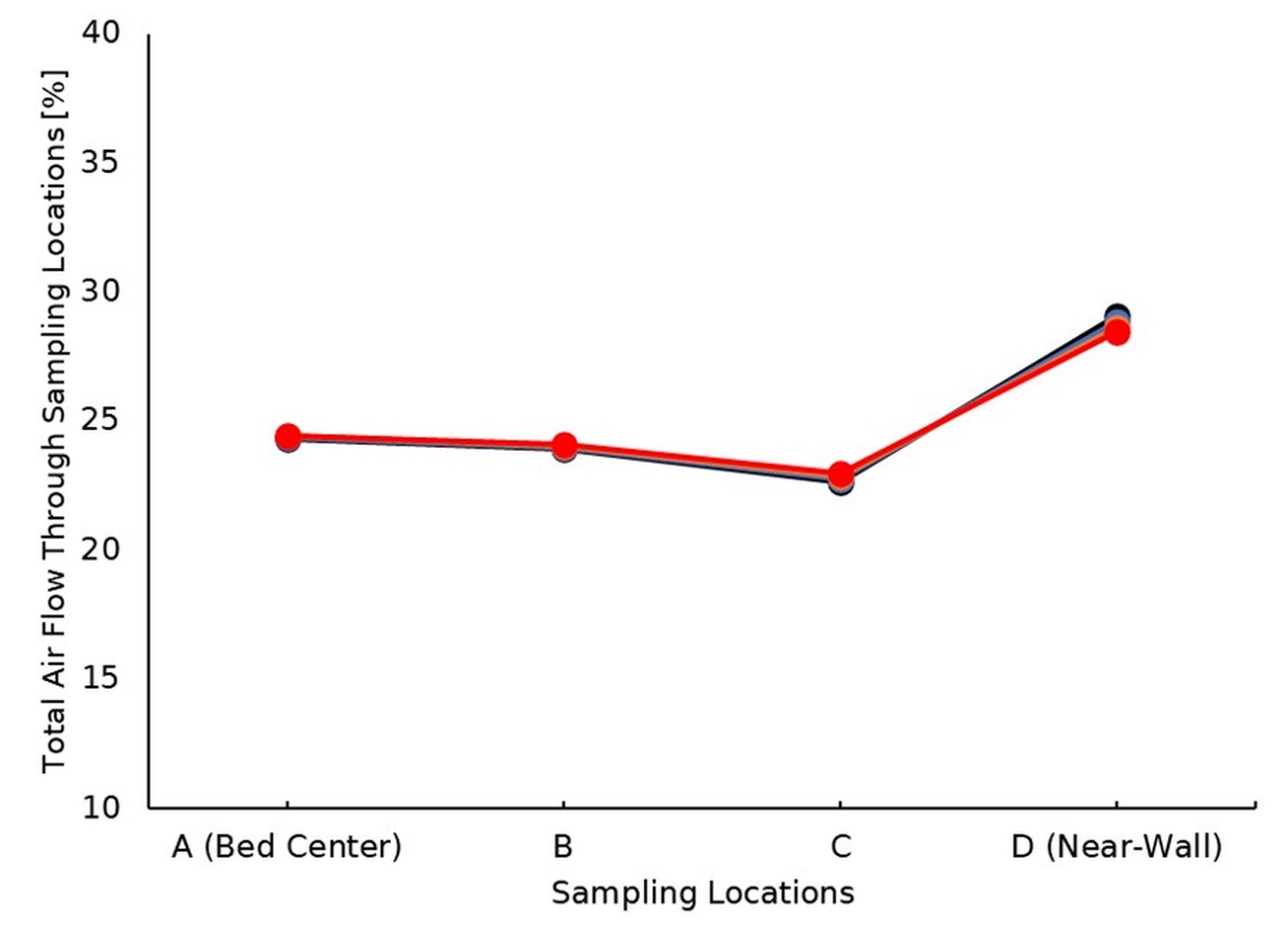


Figure D18: Distribution of total air flow through the outlet sampling locations for each injection air flux investigated: 1 cm s^-1^ (black line, Base Case), 1.5 cm s^-1^ (blue line, medium air flux), 2 cm s^-1^ (orange line, high air flux), and 3 cm s^-1^ (red line, maximum air flux).


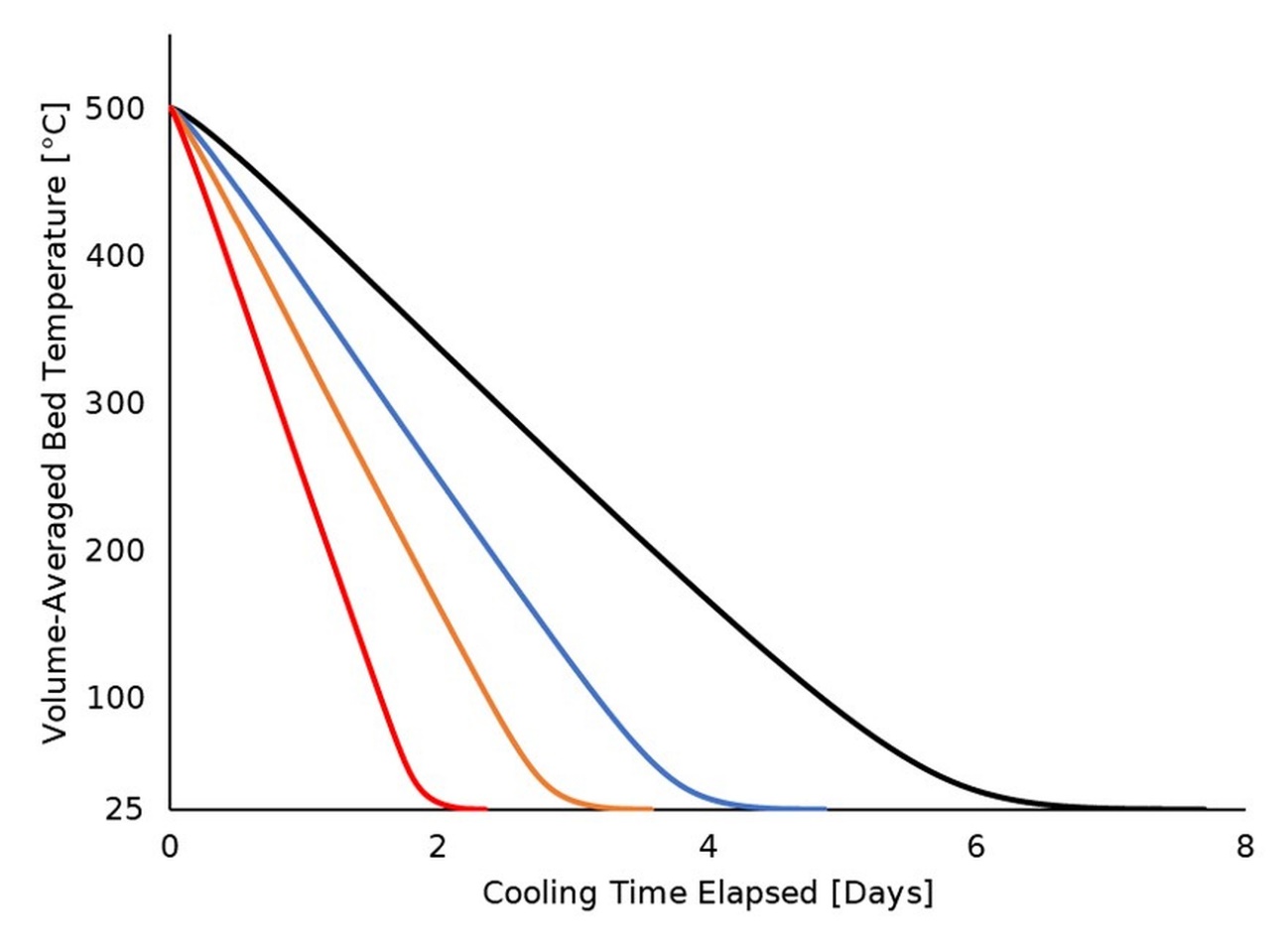


Figure D19: Transient volume-averaged bed temperatures for each injection air flux investigated: 1 cm s^-1^ (black line, Base Case), 1.5 cm s^-1^ (blue line, medium air flux), 2 cm s^-1^ (orange line, high air flux), and 2490 kg m^-3^ (red line, maximum air flux).


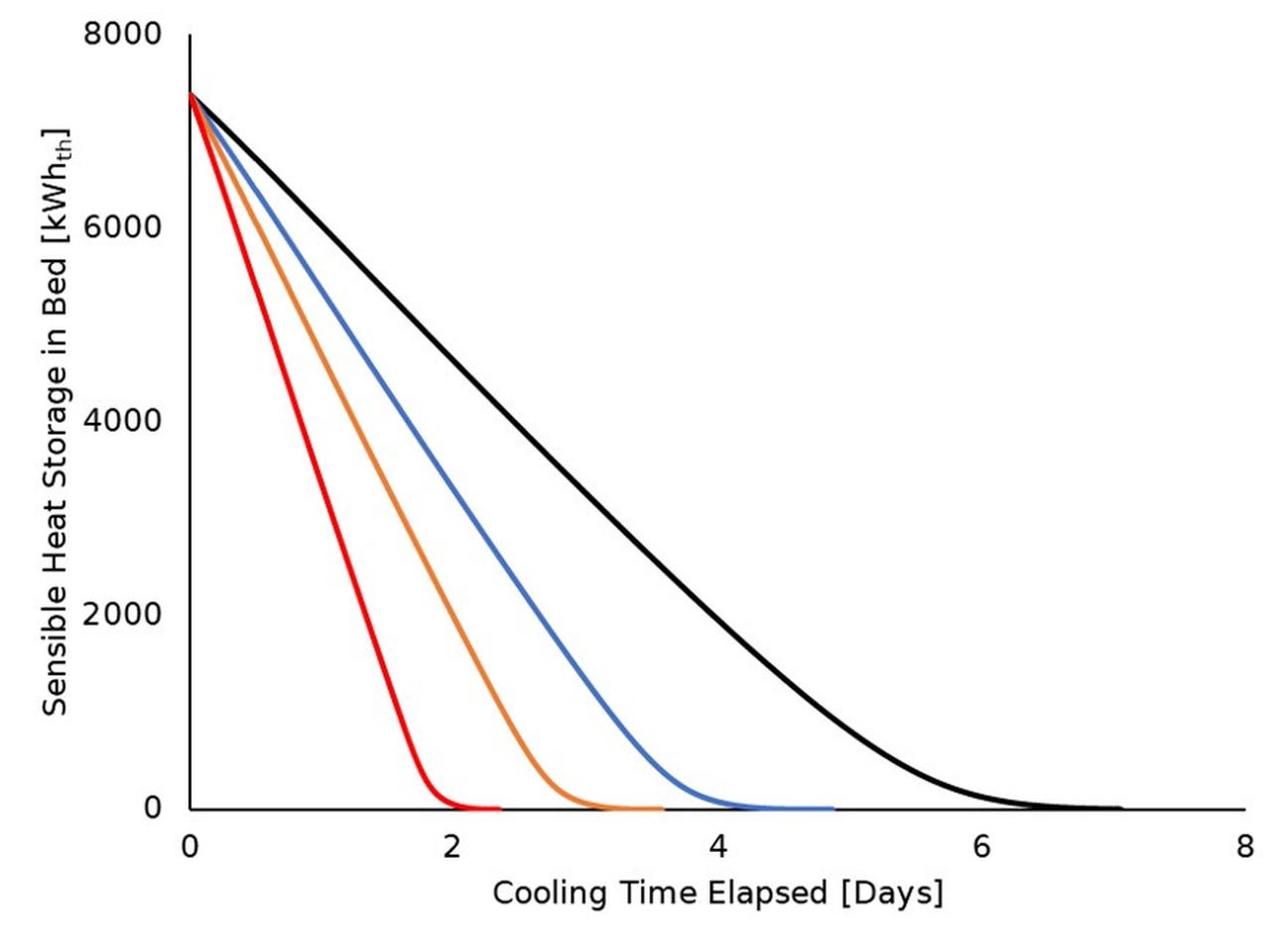


Figure D20: Transient sensible heat storage in the bed for each injection air flux investigated: 1 cm s^-1^ (black line, Base Case), 1.5 cm s^-1^ (blue line, medium air flux), 2 cm s^-1^ (orange line, high air flux), and 2490 kg m^-3^ (red line, maximum air flux).


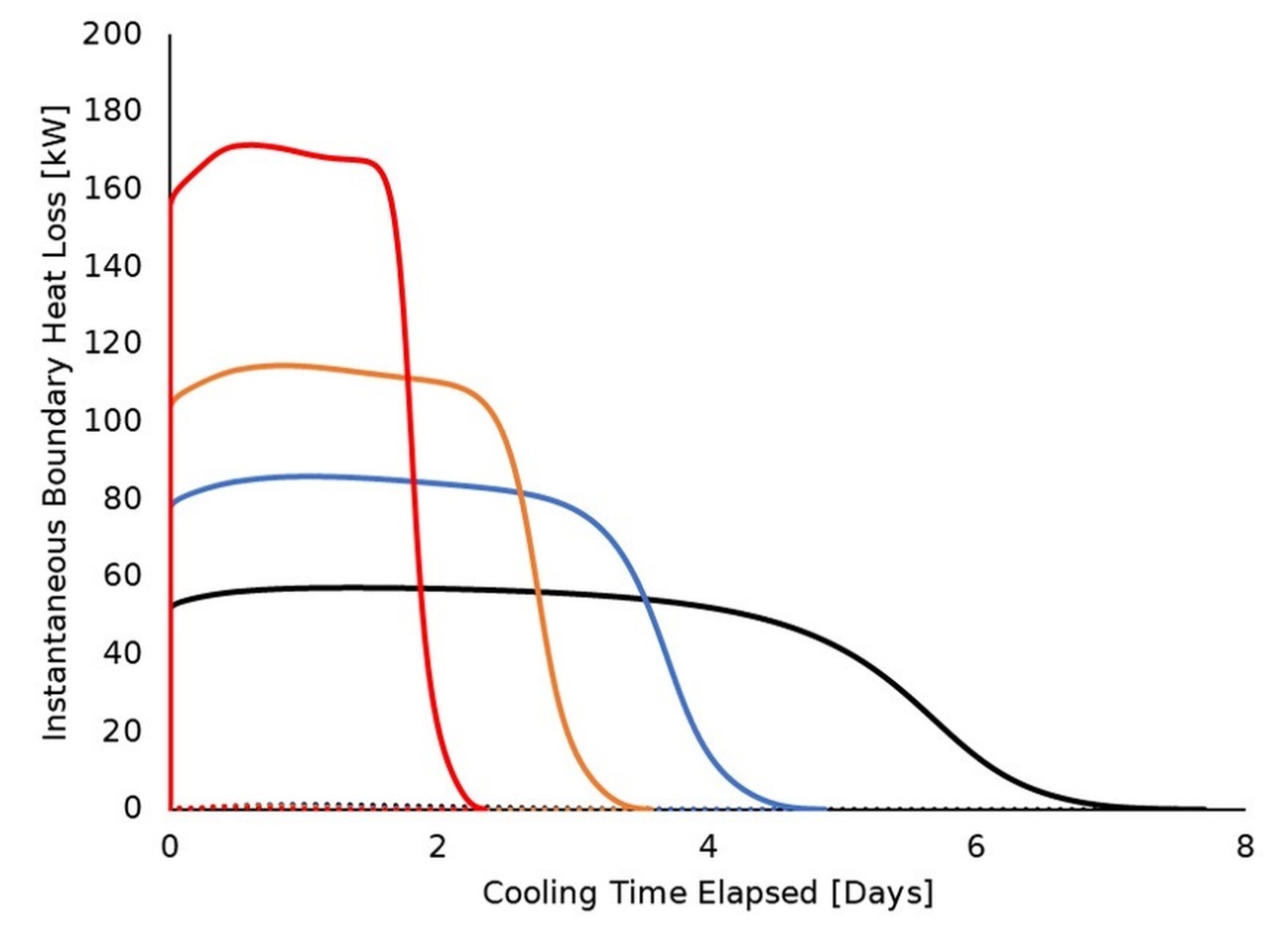


Figure D21: Transient boundary heat losses for each injection air flux investigated: 1 cm s^-1^ (black line, Base Case), 1.5 cm s^-1^ (blue line, medium air flux), 2 cm s^-1^ (orange line, high air flux), and 2490 kg m^-3^ (red line, maximum air flux). Solid lines indicate outlet heat losses, dotted lines are wall heat losses.

# Supplement References

ANSYS Inc. (2020). *ANSYS® Student Fluent 2020 R1/R2, Help System, User’s Guide*.

Baud, G., Salvador, S., Debenest, G., & Thovert, J. F. (2015). New Granular Model Medium to Investigate Smoldering Fronts Propagation-Experiments. *Energy and Fuels*, *29*(10), 6780–6792. https://doi.org/10.1021/acs.energyfuels.5b01325

Celik, I. B., Ghia, U., Roache, P. J., Freitas, C. J., Coleman, H., & Raad, P. E. (2008). Procedure for Estimation and Reporting of Uncertainty Due to Discretization in CFD Applications. *Journal of Fluids Engineering*, *130*(7), 078001. https://doi.org/10.1115/1.2960953

Martins, M. F., Salvador, S., Thovert, J. F., & Debenest, G. (2010). Co-current combustion of oil shale - Part 2: Structure of the combustion front. *Fuel*, *89*(1), 133–143. https://doi.org/10.1016/j.fuel.2009.06.040

Pironi, P., Switzer, C., Gerhard, J. I., Rein, G., & Torero, J. L. (2011). Self-sustaining smoldering combustion for NAPL remediation: Laboratory evaluation of process sensitivity to key parameters. *Environmental Science and Technology*, *45*(7), 2980–2986. https://doi.org/10.1021/es102969z

Zanoni, M. A. B., Torero, J. L., & Gerhard, J. I. (2017). Determination of the interfacial heat transfer coefficient between forced air and sand at Reynold’s numbers relevant to smouldering combustion. *International Journal of Heat and Mass Transfer*, *114*, 90–104. https://doi.org/10.1016/j.ijheatmasstransfer.2017.06.020

Zanoni, M. A. B., Torero, J. L., & Gerhard, J. I. (2019). The role of local thermal non-equilibrium in modelling smouldering combustion of organic liquids. *Proceedings of the Combustion Institute*, *37*(3), 3109–3117. https://doi.org/10.1016/j.proci.2018.05.177

Zanoni, M. A. B., Wang, J., & Gerhard, J. I. (2021). Understanding pressure changes in smouldering thermal porous media reactors. *Chemical Engineering Journal*, *412*, 128642. https://doi.org/10.1016/j.cej.2021.128642

1. Department of Civil and Environmental Engineering, The University of Western Ontario, Canada

   2 Department of Mechanical and Materials Engineering, The University of Western Ontario, Canada

   ^3^ Savron Solutions, Canada

   ^4^ Corresponding author. Address: The University of Western Ontario, Spencer Engineering Building, London, ON N6A 5B9, Canada. Email: jgerhard@uwo.ca [↑](#footnote-ref-1)
