# Supplementary material for: Understanding, controlling and optimising the cooling of waste thermal treatment beds including STARx Hottpads
Source: Waste Manag Res. 2022 Mar 21;40(9):1390–401. doi: 10.1177/0734242X221076308 (PMC9393652; doi:10.1177/0734242X221076308)
Supplement: sj-docx-2-wmr-10.1177_0734242X221076308 – Supplemental material for Understanding, controlling and optimising the cooling of waste thermal treatment beds including STARx Hottpads [file sj-docx-2-wmr-10.1177_0734242X221076308.docx]

Understanding, Controlling and Optimizing the Cooling of Thermal Waste Treatment Beds including STARx Hottpads

- Video Titles + Descriptions for Supplementary Material -

Ryan B Morales^[[1]](#footnote-1)^, Christopher T DeGroot^2^, Grant Scholes^3^, and Jason I Gerhard^1,^4

Submitted to Waste Management & Research

**SUPPLEMENT B: MODEL VALIDATION DETAILS**

***Column Experiment Cooling Phase Simulation***

🡪 VIDEO 1:

Title: “Model_Validation-Temperature_Distribution”

Description: Evolution of bed temperature distribution during the simulation of the cooling phase of a column reactor smouldering experiment

**SUPPLEMENT D: COMPILATION OF SIMULATION RESULTS**

***Base Case Simulation:***

🡪 VIDEO 2:

Title: “Base_Case-Temperature_Distribution”

Description: : Evolution of bed temperature distribution throughout the Base Case cooling phase simulation.

🡪 VIDEO 3:

Title: “Base_Case-Pneumatic_Conductivity_Distribution”

Description: Evolution of bed pneumatic conductivity distribution throughout the Base Case cooling phase simulation.

***Initial Volume-Averaged Bed Temperature Simulations:***

🡪 VIDEO 4:

Title: “Temp_750C-Temperature_Distribution”

Description: Evolution of bed temperature distribution throughout the 750°C cooling phase simulation.

🡪 VIDEO 5:

Title: “Temp_750C-Pneumatic_Conductivity_Distribution”

Description: Evolution of bed pneumatic conductivity distribution throughout the 750°C cooling phase simulation.

🡪 VIDEO 6:

Title: “Temp_1000C-Temperature_Distribution”

Description: Evolution of bed temperature distribution throughout the 1000°C cooling phase simulation

🡪 VIDEO 7:

Title: “Temp_1000C-Pneumatic_Conductivity_Distribution”

Description: Evolution of bed pneumatic conductivity distribution throughout the 1000°C cooling phase simulation

🡪 VIDEO 8:

Title: “Temp_1250C-Temperature_Distribution”

Description: Evolution of bed temperature distribution throughout the 1250°C cooling phase simulation

🡪 VIDEO 9:

Title: “Temp_1250C-Pneumatic_Conductivity_Distribution”

Description: Evolution of bed pneumatic conductivity distribution throughout the 1250°C cooling phase simulation

***Initial Bed Temperature Distribution Simulations:***

🡪 VIDEO 10:

Title: “Dist_Homogeneous-Temperature_Distribution”

Description: Evolution of bed temperature distribution throughout the Homogeneous initial temperature distribution cooling phase simulation

🡪 VIDEO 11:

Title: “Dist_Homogeneous-Pneumatic_Conductivity_Distribution”

Description: Evolution of bed pneumatic conductivity distribution throughout the Homogeneous initial temperature distribution cooling phase simulation

🡪 VIDEO 12:

Title: “Dist_Vertical_Gradient-Temperature_Distribution”

Description: Evolution of bed temperature distribution throughout the Vertical Gradient initial temperature distribution cooling phase simulation

🡪 VIDEO 13:

Title: “Dist_Vertical_Gradient-Pneumatic_Conductivity_Distribution”

Description: Evolution of bed pneumatic conductivity distribution throughout the Vertical Gradient initial temperature distribution cooling phase simulation

🡪 VIDEO 14:

Title: “Dist_Horizontal_Gradient-Temperature_Distribution”

Description: Evolution of bed temperature distribution throughout the Horizontal Gradient initial temperature distribution cooling phase simulation

🡪 VIDEO 15:

Title: “Dist_Horizontal_Gradient-Pneumatic_Conductivity_Distribution”

Description: Evolution of bed pneumatic conductivity distribution throughout the Horizontal Gradient initial temperature distribution cooling phase simulation

***Porous Bed Bulk Density Simulation:***

🡪 VIDEO 16:

Title: “Bulk_Density_415-Temperature_Distribution”

Description: Evolution of bed temperature distribution throughout the 415 kg m^-3^ cooling phase simulation

🡪 VIDEO 17:

Title: “Bulk_Density_415-Pneumatic_Conductivity_Distribution”

Description: Evolution of bed pneumatic conductivity distribution throughout the 415 kg m^-3^ cooling phase simulation

🡪 VIDEO 18:

Title: “Bulk_Density_830-Temperature_Distribution”

Description: Evolution of bed temperature distribution throughout the 830 kg m^-3^ cooling phase simulation

🡪 VIDEO 19:

Title: “Bulk_Density_830-Pneumatic_Conductivity_Distribution”

Description: Evolution of bed pneumatic conductivity distribution throughout the 830 kg m^-3^ cooling phase simulation

🡪 VIDEO 20:

Title: “Bulk_Density_2490-Temperature_Distribution”

Description: Evolution of bed temperature distribution throughout the 2490 kg m^-3^ cooling phase simulation

🡪 VIDEO 21:

Title: “Bulk_Density_2490-Pneumatic_Conductivity_Distribution”

Description: Evolution of bed pneumatic conductivity distribution throughout the 2490 kg m^-3^ cooling phase simulation

***Injection Air Flux Simulations:***

🡪 VIDEO 22:

Title: “Air_Flux_1.5-Temperature_Distribution”

Description: Evolution of bed temperature distribution throughout the 1.5 cm s^-1^ cooling phase simulation.

🡪 VIDEO 23:

Title: “Air_Flux_1.5-Pneumatic_Conductivity_Distribution”

Description: Evolution of bed pneumatic conductivity distribution throughout the 1.5 cm s^-1^ cooling phase simulation.

🡪 VIDEO 24:

Title: “Air_Flux_2.0-Temperature_Distribution”

Description: Evolution of bed temperature distribution throughout the 2.0 cm s^-1^ cooling phase simulation.

🡪 VIDEO 25:

Title: “Air_Flux_2.0-Pneumatic_Conductivity_Distribution”

Description: Evolution of bed pneumatic conductivity distribution throughout the 2.0 cm s^-1^ cooling phase simulation.

🡪 VIDEO 26:

Title: “Air_Flux_3.0-Temperature_Distribution”

Description: Evolution of bed temperature distribution throughout the 3.0 cm s^-1^ cooling phase simulation.

🡪 VIDEO 27:

Title: “Air_Flux_3.0-Pneumatic_Conductivity_Distribution”

Description: Evolution of bed pneumatic conductivity distribution throughout the 3.0 cm s^-1^ cooling phase simulation.

1. Department of Civil and Environmental Engineering, The University of Western Ontario, Canada

   2 Department of Mechanical and Materials Engineering, The University of Western Ontario, Canada

   ^3^ Savron Solutions, Canada

   ^4^ Corresponding author. Address: The University of Western Ontario, Spencer Engineering Building, London, ON N6A 5B9, Canada. Email: jgerhard@uwo.ca [↑](#footnote-ref-1)
